# Victims, Vignettes, and Videos: Meta-Analytic and Experimental Evidence That Emotional Impact Enhances the Derogation of Innocent Victims

**DOI:** 10.1177/1088868320914208

**Published:** 2020-04-22

**Authors:** Rael J. Dawtry, Mitchell J. Callan, Annelie J. Harvey, Ana I. Gheorghiu

**Affiliations:** 1 University of Essex, Colchester, UK; 2 University of Bath, UK; 3 Anglia Ruskin University, Cambridge, UK; 4 University of Portsmouth, UK

**Keywords:** victim derogation, responses to victimization, just-world theory, emotional impact, meta-analysis

## Abstract

Research during the 1960s found that observers could be moved enough by an innocent victim’s suffering to derogate their character. However, recent research has produced inconsistent evidence for this effect. We conducted the first meta-analysis (*k* = 55) of the experimental literature on the victim derogation effect to test the hypothesis that it varies as a function of the emotional impactfulness of the context for observers. We found that studies which employed more impactful contexts (e.g., that were real and vivid) reported larger derogation effects. Emotional impact was, however, confounded by year of appearance, such that older studies reported larger effects and were more impactful. To disentangle the role of emotional impact, in two primary experiments we found that more impactful contexts increased the derogation of an innocent victim. Overall, the findings advance our theoretical understanding of the contexts in which observers are more likely to derogate an innocent victim.

The various ways observers react to others’ undeserved pain, suffering, and misfortune have long intrigued social scientists (see, for example, Heider, 1958; Lerner, 1980; Ryan, 1971). Perhaps one of the most striking of these reactions is the tendency for observers to, at times, derogate the victim. That is, rather than showing sympathy and compassion, observers may respond to an innocent victim by devaluing their character. In the first experimental demonstration of this phenomenon, Lerner and Simmons (1966) asked participants to view a live video feed (in reality a recording) of a confederate completing a learning task and receiving electric shocks for responding incorrectly, to which she reacted with expressions of pain and anguish. They found that participants who believed that the learner would continue to receive painful shocks in a subsequent phase of the experiment evaluated her character *less* favorably (e.g., as less likable) than did participants who learned that her ordeal had ended.

But why would seemingly otherwise decent people derogate an innocent victim? Indeed, the capacity for people to derogate an innocent victim is puzzling because, by any commonly accepted standards or norms, people ought not to devalue someone’s character for negative outcomes brought about by chance or factors beyond their control (Dawtry et al., 2018; Weiner, 1995). The need to believe in a just world emerged as one explanation (Lerner, 1977, 1980). Specifically, just-world theory posits that derogating an innocent victim enables the observer to maintain the functional belief that the world is a just, fair, and nonrandom place in which people get what they deserve and deserve what they get. According to this perspective, believing in a just world is important for pursuing long-term goals with confidence (Callan et al., 2009; Hafer, 2000b; Lerner, 1977), and may be threatened by the knowledge that someone is suffering through little fault of their own. Derogating an innocent victim therefore helps people sustain the self-regulatory benefits of assuming that the world is a just and fair place, because doing so effectively removes the injustice—in a just world, bad things happen *only* to “bad” people.

Just-world theory is widely known and has had far-reaching influence on research and theorizing in social psychology and beyond. The theory is featured in several prominent psychology textbooks (e.g., Aronson et al., 2013; Myers & DeWall, 2015); it continues to be invoked in news reports and popular media as a key explanation for why people reject innocent victims (e.g., Burns, 2018; Sargent, 2014; Szalavitz, 2018); it has inspired hundreds of studies into the nature, functions, and consequences of believing in a just world across both experimental (Ellard et al., 2016; Hafer & Bègue, 2005) and individual differences traditions (Furnham, 2003; Hafer & Sutton, 2016); and it helped lay the groundwork for further theorizing in social psychology, such as for system justification theory (Jost & Banaji, 1994) and terror management theory (Solomon et al., 1991).

Empirical support for just-world theory was bolstered by a series of replications of Lerner and Simmons’s (1966) experiment throughout the late 1960s and early 1970s (e.g., Kenrick et al., 1976; Lerner, 1971; Lincoln & Levinger, 1972; C. W. Simons & Piliavin, 1972; Sorrentino & Hardy, 1974; Stokols & Schopler, 1973). Seeking to examine explanations for and extensions of the victim derogation effect, these studies typically used similar procedures to Lerner and Simmons, exposing participants to a vivid, immediate, and ostensibly genuine episode of victimization. This work consistently showed that observers evaluated the victim’s character less favorably when they were suffering (vs. not suffering). For example, Lerner (1971) found that participants who believed that the learner was genuinely being shocked rated her less favorably than did participants who either believed that she was acting or would be compensated for her ordeal.

Yet, despite the theory’s reach and influence and the wave of replications of Lerner and Simmons’s (1966) experiment before 1980, recent experimental research has seemingly produced inconsistent evidence for the victim derogation effect. Although some more recent experiments have found evidence for the victim derogation effect (e.g., Carli, 1999; Carli & Leonard, 1989; Heater et al., 2002; Lea & Hunsberger, 1990), several have not (e.g., Callan et al., 2014; Harvey et al., 2014), and a few studies even demonstrated the opposite tendency, that is, *enhancement* of a victim’s character under high just-world threatening conditions (e.g., Burczyk & Standing, 1989; Callan et al., 2007; Lens et al., 2014). Thus, on one hand, research conducted in the 1960s and 1970s found robust and consistent evidence that observers could be threatened enough by an innocent victim’s suffering to derogate their character, and this early work formed the empirical bedrock on which just-world theory rests. On the other hand, research since the 1980s has seemingly found, at best, inconsistent evidence for the victim derogation effect. Of course, just-world theory is not only concerned with why people derogate innocent victims, nor is victim derogation the only psychological defense against threats to the need to believe in a just world (see Callan & Ellard, 2010; Ellard et al., 2016; Hafer & Rubel, 2015), but victim rejection is nonetheless widely viewed as a hallmark of the theory. The findings from contemporary experimental research into victim derogation therefore raise questions about the robustness and replicability of the phenomenon most commonly associated with just-world theory. Achieving a better understanding of the conditions under which victim derogation is likely to manifest is therefore of practical and theoretical importance.

What, then, might account for the apparent disparity between the early research, which generally replicated Lerner and Simmons’s (1966) initial finding, and the research post-1980 that has produced results that are more ambivalent? One straightforward explanation is that the early findings are historically and culturally specific. Conceivably, positive changes in social norms and attitudes over the past 50 years, such as through the disability rights movement (Fleischer et al., 2012) or increased societal concerns about political correctness (Fairclough, 2003), have affected how people generally respond to others who have been victimized, rendering victim derogation relatively less likely to manifest in contemporary research. Indeed, similar observations have been made for other social-psychological phenomena, such as conformity, which has declined since Asch’s (1952, 1956) seminal research in the 1950s (Bond & Smith, 1996; Perrin & Spencer, 1981).

Another potential explanation relates to methodological differences between the classic and contemporary studies. Contemporary researchers have employed a variety of stimuli and procedures to confront participants with the suffering of innocent victims, with a large number of these studies using text-based vignettes (e.g., Harvey et al., 2014; Lea & Hunsberger, 1990), and occasionally video news reports of past events or interviews with victims describing their suffering (e.g., Aguiar et al., 2008; Hafer, 2000a, 2000b). Hafer and Bègue (2005) and Lerner (2003) argued that such relatively “low impact” victimization contexts may be insufficiently threatening or emotionally engaging to elicit the derogation of victims. Furthermore, stimuli in which the victim does not clearly continue to suffer in the present are potentially less impactful (Hafer, 2000a; Lerner & Simmons, 1966), as are stimuli that entail relatively minor or trivial injustices (Hafer & Bègue, 2005). Notably, this recent research stands in stark contrast to the pre-1980 studies that placed participants in an immediate, vivid, and ostensibly real victimization context.

Drawing on dual-process theories (Chaiken & Trope, 1999), Lerner (2003; see also Lerner & Clayton, 2011) theorized that “low impact” victimization contexts present little threat to the need to believe in a just world; they provoke conscious, thoughtful consideration of the circumstances surrounding an episode of victimization, and of the normatively appropriate response toward them. Because people generally do not want to appear irrational or unsympathetic in front of others, given sufficient time and cognitive resources, their responses will tend to reflect conventional norms surrounding how one *should* respond to the suffering of an innocent victim (i.e., with positivity rather than derogation or blame; see Dawtry et al., 2018). In Lerner’s view, derogation is unlikely to appear in low-impact contexts because threat to the need to believe in a just world is low, cognitive resources are relatively unconstrained, and impression management concerns are likely to dominate. When confronted with an emotionally involving and real episode of victimization, however, emotion-driven strategies serving to defend the belief in a just world from immediate threat, such as victim derogation, are relatively more likely to emerge.

But what features of a victimization context might contribute to the level of “emotional impact” experienced by observers? As we alluded to above, one important methodological feature that is likely related to emotional impact is the medium by which observers are exposed to a victimization context, for example, whether they read a text vignette (e.g., Harvey et al., 2014), view recorded or live video footage of the event (e.g., Lerner & Simmons, 1966), or see a victim describing their experiences during a video interview (e.g., Warner et al., 2012). This is because the medium partially determines and constrains other attributes of the stimuli that theoretically cause emotional impact, including the vividness with which the events are conveyed, and their perceived veracity, severity, and proximity.

## Vividness

Vividness refers to the perceptual immediacy and intensity with which a victim’s suffering is depicted, or the extent to which stimuli create powerful mental images. According to Nisbett and Ross (1980), vivid stimuli are “(a) emotionally interesting, (b) concrete and imagery provoking, and (c) proximate in a sensory, temporal or spatial way” (p. 45). Much evidence suggests that attributes related to vividness, such as image size (Codispoti & De Cesarei, 2007), motion (R. F. Simons et al., 1999), and stereoscopic depth (2D vs. 3D film; Rooney et al., 2012), moderate perceivers’ emotional response to stimuli. R. F. Simons et al. (1999), for example, found that moving (vs. static) versions of emotion-relevant stimuli evoked stronger self-reported emotions, as well as physiological arousal indexed via electrodermal activity and heart rate. Third-person text vignettes, which generally involve an abstract and un-emotive description of an episode of victimization, arguably possess relatively low vividness. Alternatively, witnessing an injustice unfold firsthand or via video is presumably highly vivid, insofar the event is directly experienced rather than imagined based on an after-the-fact, secondhand description. Correspondingly, video presentations elicit stronger self-reported emotion and engagement than when the same information is presented via text alone (Koehler et al., 2005; Yadav et al., 2011). Furthermore, research on the impact of “gruesome evidence” on jury decision making suggests a link between vividness, emotion, and justice judgments (Bright & Goodman-Delahunty, 2006; Oliver & Griffitt, 1976; Whalen & Blanchard, 1982). Bright and Goodman-Delahunty (2006), for example, found that mock-jurors presented with gruesome photographs of a victim’s injuries (vs. no photos) reported more intense emotions, and were more likely to convict the defendant. Vivid victimization contexts (e.g., presented through video) may be more emotionally arousing than those that lack vividness (e.g., text vignettes) partly because they facilitate a stronger empathic response; research has shown that directly witnessing pain and distress in others triggers aversive emotional arousal and activates neural regions linked to the experience of such states (e.g., Decety & Jackson, 2006; Eisenberg & Miller, 1987; Preston & De Waal, 2002).

## Veracity

Lerner (2003) and others (e.g., Hafer & Bègue, 2005) argued that real or ostensibly real injustices are more emotionally arousing than hypothetical situations, insofar as observers may perceive hypothetical scenarios as unrealistic and irrelevant to their justice concerns. Indeed, victim derogation did not occur in the Lerner and Simmons (1966) “shock victim” situation when participants were made aware that the events were a role-play (Lerner, 1971; C. W. Simons & Piliavin, 1972). Hypothetical suffering is perhaps unlikely to evoke a strong emotional response and establish a strong motivational imperative to defend just-world beliefs because, strictly speaking, no injustice has *actually* transpired. Relatedly, evidence shows that ostensibly real instances of suffering provoke stronger self-reported emotion in observers (Mendelson & Papacharissi, 2007), as well as greater physiological arousal (Fan & Han, 2008; Geen, 1975; Gu & Han, 2007), compared with hypothetical or fictional suffering. Gu and Han (2007), for example, found that neural activity associated with empathy for pain was reduced when participants were shown cartoons of hands in a painful condition, versus photographs of real hands in the same painful condition. As mentioned previously, veracity is presumably determined in part by the stimulus medium: video footage (real or ostensibly real), for example, potentially possesses greater veracity than text vignettes, insofar as such footage is less easily dismissed as fictional or contrived.

## Proximity

It is also assumed that people react more strongly to events that are closer to the self in space and time (e.g., Liberman et al., 2007; Trope & Liberman, 2010). The notion that the impact of negative events becomes less acute as time passes (Suh et al., 1996) is implied in the folk saying “time is a great healer,” and injustices (e.g., terrorism, natural disasters) occurring in faraway places often seem to provoke relatively muted responses. Proximity appears to affect observers’ emotional response, such that physiological arousal decreases with increases in the spatial distance (Davis et al., 2011; Mühlberger et al., 2008), as well reductions in the physical size (Codispoti & De Cesarei, 2007), of unpleasant or threatening stimuli. Recent evidence further suggests that proximity modulates empathic processes. Lomoriello et al. (2018) measured event-related potentials (ERPs) across fronto-parietal and centro-parietal regions in response to images of neutrally and painfully stimulated faces that appeared either spatially close or distant. Empathy-linked ERP responses across both regions were greater among participants exposed to faces that appeared close, versus those who saw relatively distant faces. Relatedly, research shows that people seek to physically distance themselves from stimuli that are potentially threatening to the need to believe in a just world, such as salient charity appeals (Pancer, 1988; Pancer et al., 1979). Episodes of victimization that are relatively more proximal to the observer, then, are likely to elicit a stronger emotional response than those that are more distant in time or space, and correspondingly, distancing oneself from injustice may alleviate negative emotions associated with injustice.

## Severity

Emotional responses toward episodes of victimization are also potentially influenced by perceived severity, which encompasses both the immediate cause of suffering (e.g., violence, rape, theft, illness, or accident) and the nature and extent of the consequences suffered by a victim. Those victimization contexts perceived to be most severe presumably involve events that cause very acute physical and psychological suffering in the moment they occur, which continues (or threatens to continue) into the foreseeable future. Violent crimes, for example, are presumably perceived as more severe than thefts or minor accidents, insofar as the former involve greater immediate (e.g., physical pain, extreme negative emotions) and ongoing suffering (e.g., psychological trauma, life-changing injuries). This analysis echoes research showing that the nature and intensity of negative stimuli impacts perceivers’ emotional response; for example, Bradley et al. (2001) found that, in comparison to other unpleasant images (accidents, contamination, illness, loss), specifically violent and physically threatening images (attacking humans and animals, mutilated bodies) elicited stronger self-reported emotional arousal and changes in electrodermal activity. Furthermore, severity modulates activation of brain regions linked to empathy for others’ pain (Avenanti et al., 2005; Saarela et al., 2006); Saarela et al. (2006), for example, observed stronger activation in empathy-linked brain regions (anterior insular and anterior cingulate cortices) in response to faces rated as expressing more versus less intense pain. A study reported by Feigenson et al. (2001) suggests that stronger emotions evoked by severe suffering impact on victim evaluations; more (vs. less) severe consequences for a victim elicited stronger self-reported emotional arousal among mock-jurors, and in turn, higher victim blaming. More generally, the notion that more severe instances of suffering pose a greater threat to justice than those that are less severe is a central assumption in just-world research, and correspondingly, many studies operationalize injustice in this way (e.g., Callan et al., 2007; Harvey et al., 2014; Lerner & Simmons, 1966).

The foregoing analysis suggests that emotional impact is likely to be higher when researchers employ video presentations as opposed to text-based stimuli, and both video and text stimuli will be more impactful to the extent that an episode of more severe victimization is proximal, real, and vividly depicted. Text-based vignettes necessarily describe episodes of victimization occurring sometime in the past, whereas closed-circuit television (CCTV) footage may plausibly show an episode of victimization occurring in the here-and-now, as in Lerner and Simmons (1966). Similarly, whereas observers may easily dismiss text vignettes as fictional, events that are directly witnessed, either in person or via video footage, are presumably more believable. On a more nuanced level, text scenarios framed as news reports purportedly describe real-world events (e.g., Callan et al., 2007), so perhaps possess greater relevance to the real world than those presented in plain text, and video footage of the moment victimization occurred might be more vivid and intense than a victim’s retelling of the events in an interview.

In sum, we chose to focus on vividness, veracity, proximity, and severity for three reasons. First, prior research and theorizing suggests that these properties are positively related to the emotional impact of negative stimuli in general, including depictions of others’ suffering. Second, these properties are related to justice-specific responses, such as perpetrator punishment (vividness; Bright & Goodman-Delahunty, 2006), physical distancing from injustice (proximity; Pancer, 1988; Pancer et al., 1979), and evaluations of victims (severity, veracity; Feigenson et al., 2001; Lerner, 1971; C. W. Simons & Piliavin, 1972). Third, these properties are dependent on, and constrained by, the medium by which participants are exposed to an injustice, and what participants are lead to believe about its provenance. The stimulus medium, then, provides a clearly defined and objective proxy for the emotional impactfulness of stimuli employed in experimental research on victim derogation.

Anecdotally, much research pre-1980 seems to fall toward the high-impact stimulus end of the spectrum. Participants in these studies were directly exposed, in the here-and-now, to a vivid and apparently real episode of suffering (e.g., Kenrick et al., 1976; Lerner, 1971; Lerner & Simmons, 1966; C. W. Simons & Piliavin, 1972). Alternatively, the tendency to employ text-based scenarios in victim derogation research conducted post-1980 places most recent research toward the low-impact end of the spectrum. Text vignettes are inevitably a relatively low-impact method insofar as little effort is made to convince participants that such scenarios, often involving a secondhand description of a past victimization, involve ongoing adverse consequences for the victim.

## Current Research

It is important to highlight that the foregoing observations of an apparent disparity between the classic and contemporary research on the victim derogation effect has come from cursory glances at the experimental literature rather than a systematic investigation. It is therefore not clear whether the victim derogation effect has declined over time and, if so, what factors might be associated with this decline. To date, there have been two influential qualitative literature reviews of the experimental research on people’s responses to victimization (Hafer & Bègue, 2005; Lerner & Miller, 1978) but, surprisingly, there has never been a *quantitative* synthesis of this literature. In the current research, we performed a meta-analysis (Study 1) of the available experimental research on victim derogation conducted since Lerner and Simmons’s (1966) experiment to, for the first time, (a) quantitatively summarize the experimental evidence on the victim derogation effect and (b) test the hypothesis that the victim derogation effect varies as a function of the emotional impactfulness of the stimuli used in research, with studies employing more emotionally impactful contexts demonstrating stronger victim derogation effects. We complemented our meta-analysis with two primary studies (Studies 2 and 3) to cast further light on the idea that more (vs. less) emotionally impactful stimuli lead to greater derogation of an innocent victim’s character. In these studies, we operationalized “emotional impact” in terms of the stimulus medium by which participants were exposed to an episode of victimization (e.g., actual video footage of a robbery vs. a text description of the same robbery). We also conducted validation and pilot studies to test whether more (vs. less) vivid and dynamic presentations of victimization contexts are, in fact, more emotionally impactful (i.e., are more psychologically arousing and elicit more negative affect). Across these primary studies, we aimed to recruit large enough sample sizes to achieve at least 80% power to detect “small-to-medium” effect sizes (see details within individual studies). To our knowledge, this work is the first to directly investigate the role of stimulus medium in determining the emotional impact of victimization contexts, and consequently, evaluations of victims.

## Study 1: Meta-Analysis

In Study 1, we conducted a meta-analysis of experimental research on the derogation of innocent victims from Lerner and Simmons’s (1966) study onward. From Lerner’s (2003) theorizing, we predicted that experiments using relatively more emotionally impactful stimuli would observe larger victim derogation effects.

### Method

#### Inclusion criteria

To keep the meta-analysis both faithful to the early studies and manageable in size and scope, we only included effect sizes from studies that experimentally manipulated the apparent injustice of the *outcome* for an innocent victim. All studies used between-subjects designs. Correlational studies, such as those that investigated only the association between individual differences in self-reported just-world beliefs and observers’ evaluations of a victim’s character, were not included, nor were experiments that manipulated injustice via the attributes or behavior of the victims themselves (e.g., manipulation of in-group vs. out-group victims). Although these experiments may manipulate perceived injustice, they do so by making the victim seem more or less deserving of a particular outcome, rather than by varying the outcome itself. Because varying a victim’s attributes or behavior may contribute to differences in how they are evaluated irrespective of perceived injustice (i.e., injustice and victim evaluations are confounded), these studies were deemed unsuitable for inclusion.

Researchers have operationalized threats to the need to believe in a just world in a variety of ways, including the protractedness of a victim’s suffering (Lerner & Simmons, 1966), punishment of harm-doers (whether or not a harm-doer is punished; Callan et al., 2014; Hafer, 2000a), and procedural injustice (whether a person is treated fairly or unfairly; Skarlicki et al., 1998; Skarlicki & Turner, 2014). Contemporary researchers have also examined reactions toward a wide range of victim groups and contexts, including victims of rape or violent crime (e.g., Carli, 1999; Warner et al., 2012), victims of accidents (e.g., Aguiar et al., 2008; Harvey et al., 2014), sufferers of serious or chronic illnesses (e.g., Correia & Vala, 2003; Lea & Hunsberger, 1990), and victims of relatively mundane injustices, such as minor theft (e.g., Kozak et al., 2006; Williams, 1984). Although these studies diverge from those used in the earlier research, they share the common feature of manipulating perceived injustice via the outcome for the victim. Just-world theory posits that observers should evaluate an innocent victim’s character less favorably the greater the perceived injustice of the victim’s outcome, irrespective of the how the injustice came about. Only studies reporting explicit judgments of a victim’s character were included, and we did not include studies that examined responses to victimized social groups (e.g., national groups) as opposed to individual victims. Finally, for reasons of practicality, we included English-language papers only.

#### Literature search

**Figure 1.**
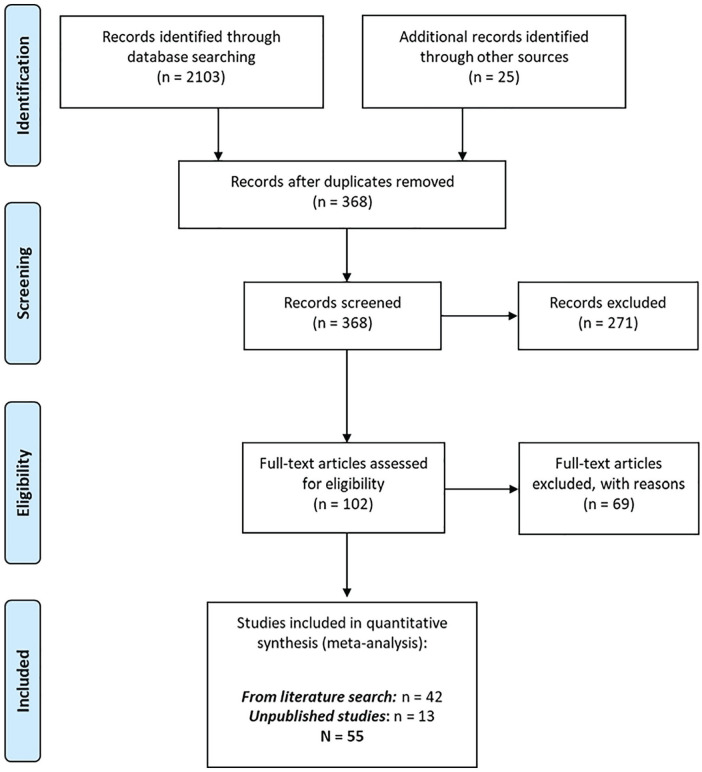
PRISMA flow diagram.

Various search strategies were used to compile a comprehensive set of experiments investigating victim derogation, including searches via several electronic databases and Google Scholar, a call for unpublished data, examination of seminal reviews on just-world theory, and personal communications. Figure 1 displays a PRISMA flowchart (Liberati et al., 2009) summarizing the literature search process.

The databases searched included Web of Science, PsycARTICLES, Medline, Education Resources Information Center (ERIC), and ProQuest Dissertations and Theses. The first three databases cover only published scholarly articles, whereas ERIC additionally includes conference papers and other materials, and ProQuest covers dissertations and theses from around the world. Each database, and Google Scholar, were searched with the phrases *victim derogation, victim denigration, victim devaluation*, and *characterological blame*. Together, these searches yielded 1,307 records (including potential duplicates across platforms). In Google Scholar, we also examined the citation record of Lerner and Simmons (1966), which was cited a total of 796 times (as of the 11th of March 2016).

The citation information of all 2,103 results was saved to a spreadsheet using the bibliographic web browser software Zotero. This allowed us to easily screen out both duplicates and items that were deemed irrelevant on the basis of document type (e.g., books, reviews, and foreign language publications), publication name (e.g., law, psychotherapy, and social work journals), or title (e.g., titles referring to self-blame or studies of victims themselves).

A total of 343 documents were earmarked for further inspection following this initial screening process. An additional 25 potentially relevant studies were identified in review papers authored by Lerner and Miller (1978) and Hafer and Bègue (2005). In total, 368 unique abstracts were examined, resulting in the retention of 102 documents (including published papers and MA/Phd theses) that were examined further. Of these, 47 were found to be unsuitable upon closer inspection (e.g., did not include relevant measures or manipulations), 17 did not report or did not provide enough statistical information to calculate an effect size (authors were contacted wherever possible), and five could not be retrieved via available channels.

Calls for unpublished data were issued to the list servers of the Society for Personality and Social Psychology (SPSP) and the International Society for Justice Research (ISJR) on the 29th of February 2016 and the 1st of March 2016, respectively, resulting in two replies and two suitable studies. A further 11 unpublished studies (including five undergraduate research projects) conducted by one of the current authors were also included. In sum, the literature search resulted in a final total of 42 fully coded studies across 33 separate documents, to which were added a further 13 unpublished studies, resulting in a final, fully coded sample of 55 studies.

#### Coding procedure

The first author independently coded all studies included in the meta-analysis, except for the 11 unpublished studies provided by the second author. These latter studies were coded by the second author in consultation with the first author. The third author independently coded 34 studies (62% of the total sample) reported in journal articles and MA/PhD theses.

##### Emotional impact

Along with recording basic background features (author name, publication year, document type, for example, journal article/PhD thesis/unpublished data), we coded the medium of the stimuli by which participants were exposed to an injustice (e.g., third-person vignette, CCTV, fictional video), which enabled us to later assign each effect an emotional impact rating. Lerner (2003) hypothesized that the victim derogation effect is likely to be stronger when the victimization context is more emotionally impactful for observers. Instead of using our own subjective judgments of which studies employed more or less emotionally impactful stimuli/contexts, we adopted a novel approach in which a sample of participants from Amazon’s Mechanical Turk (*N* = 60; 33% female; *M*_age_ = 36.15, *SD*_age_ = 12.12) rank-ordered nine different “ways of learning about others’ suffering.” The items mirrored the various types of stimuli (e.g., text vignettes, video, CCTV) represented across our sample of studies; they combined varying attributes of vividness, veracity, and proximity as they were characteristic of the actual studies included in the meta-analysis. Participants ranked the items from most (1) to least (9) emotionally arousing/impactful/upsetting (see Table 1 for the items and descriptive statistics). The items were presented to each participant in a random order.

**Table 1.** Descriptive Statistics for Rankings of the Emotional Impactfulness by Medium.

Medium	Mean rank	*SD* rank
Seeing a player being socially excluded during an online computerized ball tossing game	2.40	1.93
Watching another person suffering in a movie or fictional TV show	3.57	2.49
Reading a plain-text, secondhand (i.e., not told by the victim themselves) description of another person’s suffering	3.73	1.67
Reading a newspaper or web article describing another person’s suffering	3.88	1.74
Reading a plain-text, firsthand (i.e., recounted by the victim themselves) description of another person’s suffering	4.58	1.82
Seeing photographs showing another person suffering	5.48	1.86
Watching a recording (e.g., an interview from a TV documentary) of a person describing their own suffering firsthand	6.08	1.74
Watching another person suffering over live CCTV/camera	7.28	1.62
Watching another person suffering firsthand and in person	7.98	2.25

*Note.* CCTV = closed-circuit television.

The mean rank for each stimulus medium was matched to our coding such that each study was assigned an emotional impact score (mean emotional impactfulness ranking) according to the medium it employed; they were rescaled so higher values indicate greater perceived emotional impact of the stimulus medium. We used these emotional impact scores as our primary moderator of interest (i.e., to predict effect sizes in a meta-regression).

Although our primary interest was the moderating effect of these emotional impact scores, we also separately coded for and explored the between-study associations among stimulus vividness, veracity, spatiotemporal proximity, and the victim derogation effect. For vividness, we coded any studies using text-based stimuli as 0 (58%) and any study using audio- and/or visual-based stimuli as 1 (42%; see Table 2). For proximity, we coded any studies where the episode of victimization was clearly in the “here-and-now” for the observers (e.g., as for the Lerner & Simmons, 1966, paradigm) as 1 (20%) and any studies where the context was more spatially or temporally distant (e.g., text-based vignettes, fictional portrayals) as 0 (80%). Coding for veracity of the context posed some challenges, as it was not always clear whether researchers told the participants that the context was real or hypothetical, and in a few cases veracity formed the basis of the injustice manipulation itself. Despite this, we coded any studies where the context was clearly depicted as real as 1 (53%; e.g., Lerner & Simmons, 1966) and all other studies as 0 (inclusive of unclear and hypothetical contexts, 44%; see Table 2). We could not include veracity codes for Lerner (1971, Study 1) and C. W. Simons and Piliavin (1972) because they manipulated whether the victimization context was real or role-played (i.e., veracity was present or absent between conditions and therefore could not be coded as either at the level of study). Although Lerner’s (1971) Studies 2 and 3 also included a role-playing condition, we were able to use effect sizes from simple comparisons between other conditions that did not involve role-playing (i.e., whether the victim continued to suffer or not) for a meta-regression with veracity as the moderator. We could not meaningfully code for outcome severity in the meta-analysis because it served as the basis for many of the injustice manipulations used across studies (i.e., high severity was present or absent between experimental conditions and therefore could not be coded as either at the study level). For example, Harvey et al. (2014) varied whether a soccer player either sprained his ankle during a soccer match or sustained a serious spinal injury. Here, outcome severity is both high and low and could not be coded as either at the study level. For descriptive purposes, we also recorded the type of injustice manipulation (e.g., severity of harm, victim compensation, perpetrator punishment) and the context of the injustice (e.g., physical pain, mundane misfortune, disease/illness) (see Table 2). Finally, for factorial designs, we coded the nature of any additional independent variables crossed with the focal injustice manipulation (e.g., innocent vs. non-innocent victim; low vs. high cognitive load).

**Table 2.** Summary of Studies Included in the Meta-Analysis.

First author	Year	Source	Stimulus medium	Injustice manipulation	Injustice context	Veracity	Proximity	*n*	*g*	*SE*
Alves	2015	Journal article	Text—3rd person	Injustice present	Accident	Unclear	Distal	120	0.51	0.19
Betts	2011	Hons thesis	Text—3rd person	Severity of harm	Physical assault	Real	Distal	76	0.35	0.23
Buchanan	2008	Hons thesis	Text—3rd person	Severity of harm	Accident	Real	Distal	87	−0.53	0.22
Burczyk	1989	Journal article	Text—3rd person	Injustice present	Rape	Unclear	Distal	144	−0.64	0.17
Callan	2002	Unpublished	Video—fiction	Punishment	Physical assault	Hypothetical	Distal	34	−0.24	0.34
Callan	2003 (Study 1)	Master thesis	Video—fiction	Punishment	Physical assault	Hypothetical	Distal	45	0.19	0.3
Callan	2003 (Study 2)	Master thesis	Video—fiction	Punishment	Physical assault	Hypothetical	Distal	62	0.37	0.26
Callan	2003 (Study 3)	Master thesis	Video—fiction	Punishment	Physical assault	Hypothetical	Distal	60	−0.02	0.26
Callan	2003 (Study 4)	Master thesis	Video—interview	Compensation	Disease/illness	Real	Distal	44	0.36	0.3
Callan	2004a	Unpublished	Video—fiction	Prolonged suffering	Physical assault	Hypothetical	Distal	37	0.09	0.33
Callan	2004b	Unpublished	Video—interview	Prolonged suffering	Disease/illness	Real	Distal	27	−0.69	0.4
Callan	2004c	Unpublished	Text—1st person	Compensation	Nonviolent crime	Real	Distal	110	0.04	0.19
Callan	2007 (Study 2)	Journal article	Text—news article	Prolonged suffering	Accident	Real	Distal	36	−0.62	0.34
Callan	2014 (Study 1)	Journal article	Text—3rd person	Punishment	Physical assault	Real	Distal	375	0.07	0.1
Carli	1989	Journal article	Text—3rd person	Injustice present	Rape	Hypothetical	Distal	135	0.38	0.17
Carli	1999	Journal article	Text—3rd person	Injustice present	Rape	Real	Distal	100	0.49	0.21
Chapman	2012	Hons thesis	Text—3rd person	Severity of harm	Physical assault	Hypothetical	Distal	160	−0.16	0.16
Cialdini	1976	Journal article	Video—CCTV	Injustice present	Physical pain	Real	Proximal	86	0.91	0.23
Correia	2003 (Study 1)	Journal article	Text—3rd person	Prolonged suffering	Disease/illness	Unclear	Distal	67	−0.06	0.25
Drake	1981 (Study 1)	PhD thesis	Text—3rd person	Injustice present	Other	Hypothetical	Distal	120	0.47	0.19
Drake	1981 (Study 2)	PhD thesis	Text—3rd person	Injustice present	Other	Hypothetical	Distal	120	−0.11	0.19
Fine	1983	Journal article	Firsthand	Procedural injustice	Other	Real	Proximal	80	0.12	0.22
Gawronski	2008	Unpublished	Text—news article	Punishment	Physical assault	Real	Distal	128	−0.36	0.18
Harvey	2012	Unpublished	Text—3rd person	Severity of harm	Accident	Real	Distal	120	0.13	0.18
Harvey	2014 (Study 4)	Journal article	Text—3rd person	Severity of harm	Accident	Unclear	Distal	263	−0.16	0.12
Harvey	2014 (Study 5)	Journal article	Text—3rd person	Severity of harm	Accident	Unclear	Distal	258	−0.19	0.12
Harvey	2014 (Study 6)	Journal article	Text—3rd person	Severity of harm	Disease/illness	Unclear	Distal	220	−0.13	0.13
Irving	2003	Hons thesis	Video—fiction	Punishment	Physical assault	Hypothetical	Distal	47	0.09	0.29
Kenrick	1976	Journal article	Video—CCTV	Injustice present	Physical pain	Real	Proximal	20	1.13	0.48
Kerr	1977 (Study 1)	Journal article	Text—3rd person	Severity of harm	Rape	Unclear	Distal	218	−0.12	0.14
Knight	1980 (Study 1)	Other	Text—news article	Injustice present	Phys. assault	Real	Distal	160	−0.24	0.17
Kozak	2006 (Study 3)	Journal article	Text—3rd person	Severity of harm	Mundane misfortune	Unclear	Distal	39	1.41	0.36
Latta	1976 (Study 1)	PhD thesis	Firsthand	Severity of harm	Mundane misfortune	Real	Proximal	64	0.5	0.25
Latta	1976 (Study 2)	PhD thesis	Firsthand	Injustice present	Mundane misfortune	Real	Proximal	32	−0.25	0.35
Lea	1990	Journal article	Text—3rd person	Injustice present	Disease/illness	Real	Distal	233	0.51	0.14
Lens	2014	Journal article	Text—1st person	Severity of harm	Rape	Unclear	Distal	79	−0.81	0.23
Lerner	1966	Journal article	Video—CCTV	Prolonged suffering	Physical pain	Real	Proximal	41	0.89	0.33
Lerner	1971 (Study 1)	Journal article	Video—CCTV	Injustice present	Physical pain	Manipulated	Proximal	29	0.87	0.42
Lerner	1971 (Study 2)	Journal article	Video—CCTV	Injustice present	Physical pain	Manipulated	Proximal	34	1.69	0.41
Lerner	1971 (Study 3)	Journal article	Video—CCTV	Injustice present	Physical pain	Manipulated	Proximal	42	0.88	0.34
Lincoln	1972	Journal article	Images	Injustice present	Physical assault	Real	Distal	90	0.57	0.22
Michniewicz	2013	Journal article	Text—3rd person	Procedural injustice	Other	Unclear	Distal	38	−0.61	0.33
Murthi	2008	Hons thesis	Text—3rd person	Severity of harm	Accident	Real	Distal	50	−0.19	0.28
Park	2015	Journal article	Cyberball	Injustice present	Mundane misfortune	Real	Distal	218	−0.26	0.14
Rubel	2016	Unpublished	Video—interview	Prolonged suffering	Disease/illness	Real	Distal	254	−0.21	0.13
C. W. Simons	1972	Journal article	Video—CCTV	Injustice present	Physical pain	Manipulated	Proximal	79	0.53	0.23
Skarlicki	1998	Journal article	Text—news article	Procedural injustice	Mundane misfortune	Real	Distal	104	0.55	0.2
Skarlicki	2014 (Study 2)	Journal article	Text—3rd person	Procedural injustice	Mundane misfortune	Hypothetical	Distal	61	0.08	0.26
Sorrentino	1974	Journal article	Video—Live CCTV	Injustice present	Physical pain	Real	Proximal	80	0.55	0.23
Stokols	1973	Journal article	Text—3rd person	Severity of harm	Rape	Real	Distal	128	0.46	0.18
Telk	2012	Master thesis	Text—3rd person	Injustice present	Disease/illness	Unclear	Distal	294	−0.03	0.13
VanDeursen	2012	Journal article	Text—3rd person	Punishment	Nonviolent crime	Unclear	Distal	87	0.5	0.22
von Wurzbach	2016	Unpublished	Text—1st person	Severity of harm	Physical assault	Real	Distal	51	−0.05	0.28
Warner	2012 (Study 4)	Journal article	Video—interview	Severity of harm	Nonviolent crime	Real	Distal	96	0.58	0.21
Williams	1984 (Study 2)	Journal article	Text—3rd person	Injustice present	Rape	Unclear	Distal	165	0.39	0.16

*Note.* CCTV = closed-circuit television.

##### Inter-coder reliability

There was acceptable inter-coder reliability (Landis & Koch, 1977) for the coding of stimulus medium (κ = .79), vividness (κ = .79), type of manipulation (κ = .65), context of injustice (κ = .66), veracity (κ = .71), proximity (κ = 1), and additional independent variable codes (κ = .75). Disagreements between coders were resolved by mutual discussion.

##### Coding of statistical information

The statistical information extracted during coding included the direction of the effect (positive, that is, higher derogation under high injustice, and vice versa; κ = .78); cell or marginal cell sizes (for simple and main effects, respectively) for low, high, and any additional levels of the injustice manipulation; and the respective means and standard deviations pertaining to all derogation measures. Where cell sizes were not given, they were estimated by dividing the reported final sample size equally across conditions. Furthermore, where it was necessary to collapse reported observations across an additional measured or manipulated variable, wherever possible, means and standard deviations were weighted by cell size. Finally, where standard deviations were not reported, we extracted test statistics (e.g., *t*-values or *F*-values and their respective degrees of freedom) pertaining to the effect or effects of interest. To check that statistical data were reliably extracted, we correlated the low- and high-injustice cell sizes, means, and standard deviations recorded by the first author with, respectively, the cell sizes, means, and standard deviations recorded by the third author. Only three of the six correlations were below *r* = 1.0, and the lowest was *r* = .95, indicating that statistical information was reliably recorded by coders in the first instance. Where discrepancies did emerge, the original document was rechecked prior to computing an effect size.

#### Computation of effect sizes

Effect sizes (Cohen’s *d*) were calculated using Shadish et al.’s (1997) “ES” software. All effects were converted to Hedges’s *g* prior to analysis, which corrects for the slight upward bias of Cohen’s *d* with small samples. A study-by-study account of how each effect size was determined is available in the supplemental materials (see osf.io/a5zcp), which also lists the specific ES algorithm used in each case (methods varied depending on the statistical information available for each study). We were unable to correct effect sizes for measurement error because the majority of studies did not report scale reliabilities.

For factorial designs (*k* = 25), effect sizes were generally based upon the main effect of the focal injustice manipulation. We took this approach because a large number (*k* = 13) did not report enough information to compute simple effects, and often there was no clear rationale for determining which simple comparison afforded the most appropriate effect (e.g., studies manipulating the gender of the victim). Four studies, in which one level of the non-focal manipulation was incompatible with our inclusion criteria, were an exception to this rule. For these studies, we based effect sizes on a particular simple comparison, while excluding simple comparisons that disagreed with our inclusion criteria and were atypical of other effects included in the analyses. Specifically, Correia and Vala (2003) and von Wurzbach (2016) crossed victim suffering (suffering vs. no suffering) with a manipulation of victim innocence. For both, we used the simple effect of suffering in the innocent (as opposed to non-innocent) victim condition. Lincoln and Levinger (1972) manipulated the privacy and consequence of participants’ ratings of the victim, in addition to victim suffering (suffering vs. no suffering). Here, we used the simple effect of suffering when responses were private and non-consequential (as opposed to public and consequential). Note that the excluded simple effects in these three studies involve conditions under which derogation is generally expected not to occur, and correspondingly, the effect of suffering on derogation was predicted to be attenuated under these conditions by the study’s authors. Finally, Michniewicz and Vandello (2013) manipulated whether an advantageous or disadvantageous outcome was procedurally fair or unfair. Here, we opted to use the effect of fairness in the disadvantaged condition only, insofar as no victimization occurred in the advantaged outcome condition. Victim derogation scores dichotomized into factorial levels across non-manipulated variables (e.g., according to participant gender or self-reported belief in a just world) were always collapsed together.

Some studies manipulated injustice across more than two levels and afforded more than one comparison fitting our inclusion criteria (*k* = 8). In these cases, we aimed to combine conditions to arrive at a single pairwise effect on the basis that conditions were sufficiently conceptually similar to warrant combining them (e.g., we combined all conditions involving a similar unjust outcome for the victim, such as ill-health). Effect sizes were calculated using *n*-weighted means and standard deviations and the summed sample size across combined conditions (Higgins et al., 2011). For example, for Lea and Hunsberger (1990), we collapsed pneumonia and cancer patient conditions into a single high-injustice condition that was compared against a (non-combined) healthy control. In four cases, we used a different approach because conditions were too dissimilar to warrant combining them directly. For example, Lerner (1971, Study 2) reported comparisons for both when the victim continued to suffer versus when she was acting, and when the victim continued to suffer versus was rewarded, both of which were suitable for inclusion despite involving very different low-injustice outcomes (i.e., role-played suffering or compensated suffering). In these cases, we calculated an effect size for both comparisons, took the average of these effects, and summed the sample size across the non-repeated conditions to arrive at a single study-level effect size.

Finally, a few studies (*k* = 5) reported results separately across multiple victim derogation measures (e.g., Callan et al., 2007; Park & Park, 2015), or separately across positively and negatively-valenced subscales of a derogation measure (e.g., Correia & Vala, 2003). Multiple derogation measures were always aggregated (we took the mean across items/scales) insofar as they were deemed adequately similar in every instance.

### Results

All studies appeared between 1966 and 2016. Sample sizes ranged from 20 to 375 (*M =* 108, *SD* = 79), and 5,947 participants were included in the analyses. Analyses were conducted using the METAFOR package (Viechtbauer, 2010) in the R statistical environment. All meta-analytic models used restricted maximum likelihood estimation (REML; Viechtbauer, 2005). The 55 studies are summarized in Table 2.

#### Overall victim derogation effect

**Figure 2.**
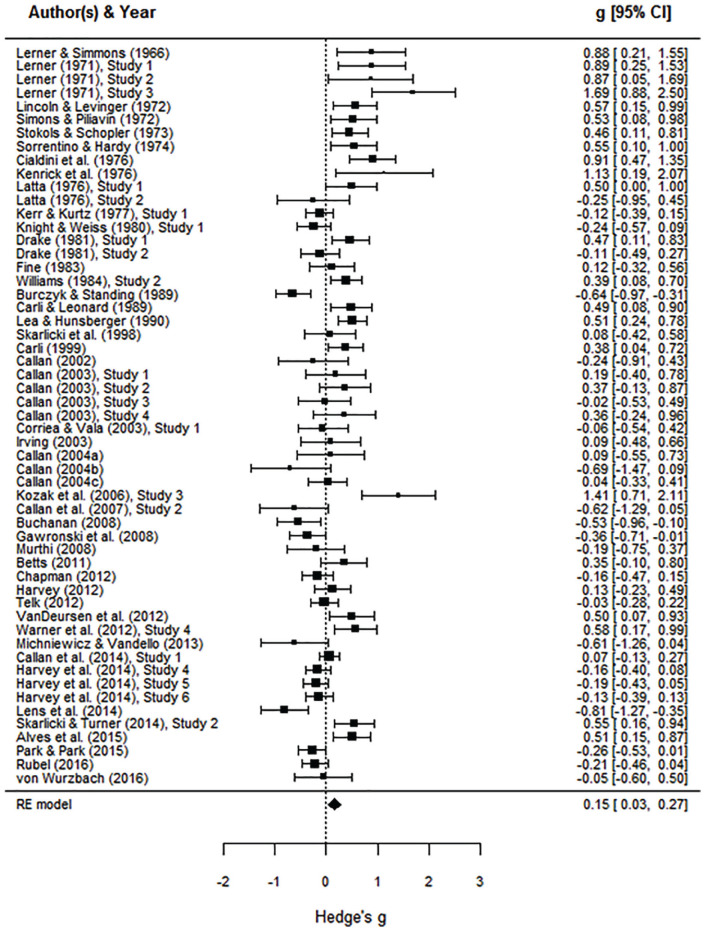
Forest plot of the overall random-effects model (*K* = 55). *Note.* Studies appear in ascending chronological order.

We first examined the overall victim derogation effect by fitting a random-effects model (e.g., Hedges, 1983; Hedges & Olkin, 1985) to the complete set of effect sizes. As shown in Figure 2, this model (*k* = 55) yielded a small overall victim derogation effect, *d* = 0.15 (*SE* = 0.06), *p* = .013, 95% confidence intervals (CIs) [0.03, 0.27], and effect sizes were significantly heterogeneous, *Q*(54) = 225.64, *p* < .001, τ^2^ = 0.15 (*SE* = 0.04), 95% CI [0.10, 0.32].^1^

#### Moderator analyses

Table 3 displays intercorrelations among the moderator variables. Consistent with our conceptual analysis, studies that were coded as more vivid, real/ostensibly real, and proximal were positively associated with those contexts our separate sample of participants ranked as more emotionally impactful. These associations are perhaps not surprising, as the mediums we asked participants to rank in terms of their emotional impactfulness combined elements of vividness, veracity, and proximity (e.g., watching someone suffer firsthand is real, highly vivid, and temporal-spatially proximal, whereas reading a secondhand description of someone’s suffering is less vivid, ambiguously real, and distant). Nonetheless, these relationships provide important evidence that vividness, veracity, and proximity correlate with perceived emotional impact. Older studies reported larger effect sizes and tended to use more real, vivid, proximal, and, therefore, more emotionally impactful stimuli. Thus, all indicators of emotional impact were confounded with year of publication/appearance.

**Table 3.** Intercorrelations Among the Moderator Variables, Study 1.

Moderator	1.	2.	3.	4.	5.	6.
1. Year of publication/appearance	—					
2. Publication status (1 = published, 0 = unpublished)	−.27*	—				
3. Sample size	.29*	.17	—			
4. Emotional impact scores	−.62**	.12	−.37**	—		
5. Vividness (1 = audiovisual, 0 = text)	−.40**	−.14	−.42**	.66**	—	
6. Proximity (1 = proximal, 0 = distal)	−.71**	.22	−.35**	.89**	.59**	—
7. Veracity (1 = real, 0 = hypothetical)	−.28*	.003	−.12	.53**	.27*	.41**

*Note. K* = 55 for all correlation except those with veracity (*k* = 53).

* *p* < .05. **p < .01.

To examine whether the size of the victim derogation effect depends on emotional impact, we fit a mixed-effects meta-regression model that included the study-level emotional impact scores (i.e., those estimated by our separate sample) as our focal moderator of the victim derogation effect. We also fit separate mixed-effect models including year of publication, vividness, veracity, and proximity as predictors. We fit mixed-effects models with each moderator separately because all the predictors were confounded (see Table 3), and the small sample size limited the statistical power available for testing multiple moderators simultaneously.

**Figure 3.**
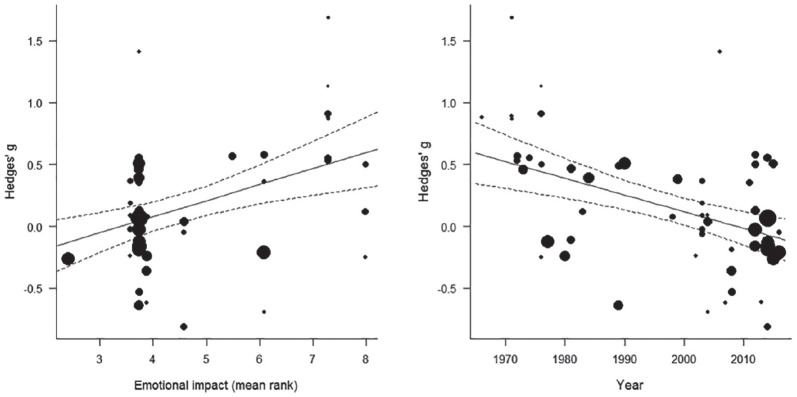
Scatterplots showing the size of the victim derogation effect by individual study plotted against emotional impact scores (left panel) and year of publication/appearance (right panel). *Note.* The sizes of the points are proportional to the inverse of the standard errors.

As shown in Table 4 and Figure 3 (left panel), emotional impact significantly moderated victim derogation, such that studies using more emotionally impactful stimuli observed larger derogation effects. Year of publication also significantly moderated victim derogation, such that older studies observed larger derogation effects (see Figure 3, right panel). Vividness and proximity, but not veracity, significantly moderated the victim derogation effect.^2^ The test for residual heterogeneity was statistically significant across all analyses.

**Table 4.** Moderator Analyses, Study 1.

Moderators	*b*	*SE*	*p*	95% CI	*Q_E_*	*p* for *Q_E_*
Emotional impact scores	0.13	0.04	<.001	[0.05, 0.21]	197.45	<.001
Year of publication/appearance	−0.01	0.004	<.001	[−0.02, −0.007]	185.09	<.001
Vividness (1 = audiovisual, 0 = text)	0.30	0.12	.014	[0.06, 0.54]	214.96	<.001
Proximity (1 = proximal, 0 = distal)	0.60	0.15	<.001	[0.31, 0.90]	184.68	<.001
Veracity (1 = real, 0 = hypothetical)	0.12	0.12	.31	[−0.11, 0.36]	204.29	<.001
Publication status (1 = published, 0 = unpublished)	0.26	0.12	.036	[0.02, 0.50]	216.87	<.001
Sample size	−0.001	0.001	.054	[−0.003, .0000]	208.20	<.001

*Note.* Each moderator was analyzed individually. Degrees of freedom for *Q_E_* equal 53 for all moderators except for veracity (*df* = 51). *Q_E_* = test for residual heterogeneity.

#### Publication bias and sensitivity analyses

To examine the data for evidence of publication bias, we fit a mixed-effects model including publication status (0 = unpublished, 1 = published) as a moderator. All studies identified as journal articles in Table 2 (*N* = 33) were coded as published, whereas all others were coded as unpublished. This model (*k* = 55) revealed that publication status significantly moderated victim derogation (see Table 4), such that published studies observed larger effects (cf. Rosenthal, 1979).

**Figure 4.**
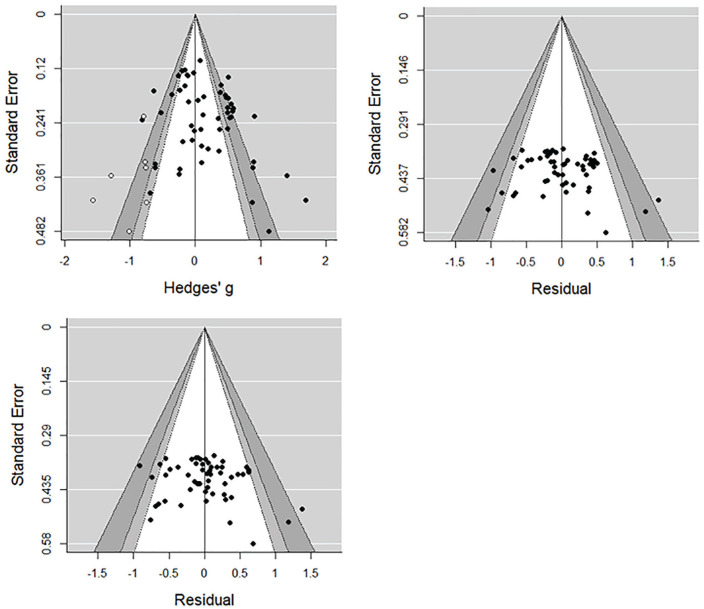
Contour-enhanced funnel plots for the overall victim derogation effect model (left panel), and mixed-effects models including emotional impact (center panel) and year of publication (right panel) as moderators. *Note.* Shaded areas represent regions of statistical significance (white—*p* > .10; light gray—*p* = .10–.05; dark gray—*p* = .05–.01; plot exterior—*p* < .01). White data points on the overall model plot (left panel) indicate effects imputed via trim-and-fill analyses.

We next plotted contour-enhanced funnel plots (see Figure 4) for the overall model and mixed-effects models including emotional impact or year of publication as moderators (see Peters et al., 2008). Effect sizes or residuals (for the overall or moderated models, respectively) were regressed onto standard errors to test for funnel plot asymmetry (Egger et al., 1997). In the overall model, effect sizes and standard errors were positively related, *z* = 2.28, *p* = .022, indicating the presence of small study effects (i.e., smaller, less precise studies contributed larger effects), but were statistically unrelated when emotional impact, *z* = 1.01, *p* = .311, or year of publication, *z* =1.42, *p* = .156, were included as moderators. Trim-and-fill analyses performed on the overall effect model estimated seven missing effects (*SE* = 4.85) on the left-hand side, shown as white data points on the funnel plot (Duval & Tweedie, 2000). Although published and less precise studies reported larger effects, it is not clear that these analyses indicate publication bias in favor of statistically significant results. Missing studies imputed via trim and fill fell in regions of statistical significance, suggesting underlying differences between smaller and larger studies rather than suppression of nonsignificant effects (Peters et al., 2008). As shown in Table 3, smaller studies were both older and used more emotionally impactful stimuli, and when we accounted for the moderating influence of either of these factors, small study effects were no longer detectable. Moreover, the relatively high proportion of unpublished (40%) and statistically nonsignificant studies (47%) goes some way to alleviating concerns regarding the influence of publication bias on the sample of studies we obtained.

Nevertheless, publication bias cannot be unambiguously ruled out under any circumstances (Vevea & Woods, 2005), and the tests for publication bias reported above should be interpreted with caution due to the relatively small sample size and high levels of unexplained heterogeneity in the data, insofar as simulations suggest these tests perform poorly under such conditions (Carter et al., 2019; Moreno et al., 2009). Hence, we sought to examine the sensitivity of the models to the presence of publication bias using weight function modeling (Hedges & Vevea, 2005; Vevea & Hedges, 1995; Vevea & Woods, 2005). This approach assumes that the likelihood that a study will be included in the meta-analytic sample is a function of its *p* value. By weighting the probability that studies with *p* values within various intervals will be sampled, an adjusted model accounting for a hypothetical pattern of publication bias, of a given magnitude and form (one- vs. two-tailed selection, favoring significant effects in only one direction vs. either direction, respectively), can be estimated. For example, considering a scenario in which only significant studies favoring the victim derogation effect were published (extreme one-tailed selection), *p* values of <.05 or >.05 would receive a weight of 1 and 0, respectively, although more fine-grained weight functions across a range of *p* values are used in practice.

Because large samples are required to reliably estimate weight functions from observed effects (Vevea & Hedges, 1995), we employed the approach described by Vevea and Woods (2005) in which a priori weight functions are used. We fit weight function models for both the overall victim derogation effect and moderation by emotional impact scores using the weights reported by Vevea and Woods (2005). Specifically, we fit weight function models pertaining to moderate and severe one-tailed selection (i.e., moderate and severe selection of victim derogation effects) and moderate and severe two-tailed selection (i.e., moderate and severe selection of victim derogation or victim enhancement effects). Analyses were performed using the *weightr* package (Coburn & Vevea, 2019) in R. As shown in Table 5, estimates for the overall victim derogation effect differed appreciably across the selection scenarios we tested. For the models assuming moderate and severe selection for *derogation* effects (one-tailed), the estimated effect size was near zero or completely reversed, respectively, whereas for the models assuming selection for derogation or enhancement effects (two-tailed), the attenuation of the estimated effect size from the no selection scenario was less severe. Which of the selection scenarios we explored best represents the actual selection scenario is, of course, unknown and left to the reader’s judgment. However, following Vevea and Woods (2005), visual inspection of the funnel plots in Figure 4 suggests a selection scenario that might more closely resemble two-tailed selection, given the presence of both significant derogation and enhancement effects. At least, a severe pattern of selection for only significant derogation effects does not appear likely given the high proportion of unpublished and nonsignificant effects included in the sample. Either way, these models suggest that, due to publication bias, the overall victim derogation effect is probably smaller than the original estimate.

**Table 5.** Sensitivity Analyses for Publication Bias Using Weight Function Modeling.

Selection condition	Overall victim derogation effect		Moderation of derogation effect by emotional impact scores	
	Estimated effect size (*g*)	Variance component	Estimated intercept	Estimated coefficient
No selection	0.153 (*SE* = 0.061)	0.149 (*SE* = 0.042)	−0.439 (*SE* = 0.180)	0.129 (*SE* = 0.038)
Moderate one-tailed	0.041	0.147	−0.551	0.133
Severe one-tailed	−0.205	0.177	−0.873	0.160
Moderate two-tailed	0.132	0.122	−0.400	0.117
Severe two-tailed	0.107	0.091	−0.350	0.102

More importantly, the estimated effects for moderation by emotional impact scores were less malleable across the selection scenarios than they were for the overall victim derogation effect. Assuming selection for derogation effects (one-tailed), the estimated moderation effect by emotional impact *increased* from the no selection scenario for both moderate and severe selection (suggesting the opposite of publication bias, assuming these selection scenarios are reasonable). Assuming selection for derogation or enhancement effects (two-tailed), the estimated moderation effect was slightly attenuated (by up to 20%) from the no selection scenario.

### Discussion

Our meta-analysis of 55 published and unpublished effect sizes over a span of 50 years revealed a small overall victim derogation effect. In line with just-world theory (Lerner, 1980), our results suggest that, overall, victims were evaluated less favorably when they posed a high (vs. low) threat to the need to believe in a just world, for example, because their suffering was greater (vs. lesser; e.g., Lerner & Simmons, 1966), was believed to be genuine (vs. role-played; e.g., Lerner, 1971), or because the harm-doer went unpunished (vs. punished; e.g., VanDeursen et al., 2012).

Meta-regressions including year of publication and emotional impact scores as moderators, however, caution that the overall effect cannot be taken at face value: older studies and those employing more emotionally impactful stimuli reported larger victim derogation effects. These moderator variables were confounded, such that older studies tended to employ more emotionally impactful stimuli than did recent studies. Furthermore, we observed excess heterogeneity both with and without the inclusion of these moderators, suggesting that additional, unaccounted-for study-level differences contribute significantly to variation in effect sizes.

Consistent with our conceptual analysis, more vivid stimuli/contexts (e.g., ostensibly live CCTV videos) and those that were real and proximal were ranked by a separate sample of participants as more emotionally impactful (although any findings with veracity should be interpreted cautiously given our difficulties coding studies as involving real vs. hypothetical contexts). These findings provide preliminary evidence that vividness, proximity, and veracity may contribute to how observers perceive the emotional impactfulness a victimization context. From these results, however, we do not know how vividness, proximity, and veracity might combine to influence the emotional impactfulness of a given episode of victimization. This was because, given the limited sample size of studies, some combinations of these factors were not represented in the data (e.g., there were no studies that were hypothetical, vivid, and proximal). What is more, we were not able to shed light on the role of outcome severity in the emotional impactfulness of an episode of victimization because we could not code several studies for severity. Thus, how vividness, proximity, veracity, and severity might combine to affect the emotional impactfulness of an episode of victimization for observers is unclear.

To address this issue empirically, we conducted a supplementary study where we asked participants to imagine being confronted with victimization scenarios that were high or low in vividness, proximity, veracity, and outcome severity and rank the potential of these scenarios for eliciting emotional impact (see Supplementary Study 1 in the supplementary materials). The results of this study complemented our meta-analysis by showing that, at least in terms of how participants imagine they would feel in these situations, victimization contexts that are vivid, real, temporally close, or have severe consequences for the victim are more emotionally impactful relative to contexts that are low in vividness, hypothetical, distal, or outcome severity.

Overall, our meta-analysis suggests that victim derogation effects have reduced since Lerner and Simmons’s (1966) original work. It is not clear, however, to what extent this reduction stems from an increased reliance on low-impact stimuli in victim derogation research over time, a change in the underlying *tendency* for people to engage in derogation (e.g., due to changing social norms), or to the influence of other, unidentified moderating variables that were not examined. If emotional impact is by proxy measuring a decline in the tendency to derogate innocent victims, then the apparent moderating role of emotional impact is potentially artifactual. Because we could not clearly disambiguate the relative contributions of emotional impact and year of appearance through our meta-analysis, we directly examined the effect of emotional impact on the derogation of victims across two primary studies.

## Study 2

In Study 2, we experimentally manipulated emotional impact by exposing participants to an episode of victimization via high- versus low-impact stimuli. Specifically, participants were presented with a victimization scenario via a third-person text vignette or a CCTV video that were otherwise matched for content. As we reported in Study 1 (see also Supplementary Study 1), videos and vignettes were judged to represent, respectively, relatively high- or low-impact stimulus mediums. Thus, varying the medium in this manner provided a valid, and practically straightforward, means of manipulating emotional impact, which corresponds to the operational definition we employed in Study 1.

Drawing on recent advances (Dawtry et al., 2018), we also tested whether the effect of emotional impact on victim derogation depends on how observers rate an innocent victim’s character—specifically, whether participant ratings of a victim’s character are made in absolute terms or against a comparative referent. Whereas absolute measures of victim derogation require respondents to make judgments in strictly absolute terms (e.g., rating a victim’s character on a scale ranging from *very negative* to *very positive*), relative measures require judgments to be made in comparison to a fixed referent, such as another person or the self (e.g., rating a person on a scale ranging from *very negatively compared to the average student* to *very positively compared to the average student*). Dawtry et al. (2018) found that victims were evaluated more negatively when character judgments were made using relative (vs. absolute) scales, and that relative judgments were only more negative than absolute (or were so to a greater degree) under conditions of high (e.g., when a victim was innocent) compared with low just-world threat (e.g., when a victim brought about their suffering through their own actions).

Dawtry et al.’s (2018) findings can be understood in terms of a tension between norms proscribing the expression of negative feelings toward victims, on one hand, and the motivation to devalue a victim, on the other. According to Dawtry et al., relative judgments obscure victim derogation behind an ostensibly rational social comparison process, thus allowing derogation to emerge in a relatively ambiguous and covert form that does not openly violate social norms or personal standards proscribing negative reactions toward innocent victims. Relative judgments may more accurately gauge the underlying *motivation* to derogate than do absolute measures, insofar as they are less prone to the influence of competing motivations to appear rational, fair-minded, and sympathetic to others’ suffering. Correspondingly, Dawtry et al. (2018) found that relative and absolute judgments of a victim diverged to a greater degree (such that relative judgments were more negative) among persons high (vs. low) in the motivation to suppress negative responses toward innocent victims.

As noted earlier, emotionally impactful stimuli (e.g., CCTV) presumably represent a stronger threat to the need to believe in a just world, consequently provoking a stronger motivation to derogate, than do low-impact stimuli (e.g., vignettes). Yet, due to the reasons outlined by Dawtry et al. (2018), absolute judgments may be less sensitive to differences in the underlying motivation to derogate under low- versus high-impact contexts, compared with relative measures.^3^ We examined this possibility in Study 2 by employing both absolute and relative judgments of the victim’s character.

In addition to Study 2, we conducted two pilot studies to ascertain whether our manipulation of stimulus medium (video vs. text) does, in fact, produce differences in emotional impact (see Supplementary Studies 2a and 2b in the supplementary materials). These studies confirmed that episodes of victimization presented in video form are more emotionally impactful than the same episodes presented as text-based vignettes.

### Method

#### Participants

A total of 561 participants (40% female; *M*_age_ = 35, *SD*_age_ = 10.49) were recruited online via Amazon’s Mechanical Turk to participate in one of four surveys.^4^ The surveys differed only in terms of the victimization context (see details below). We recruited a fixed number of participants per survey (~140 per survey, which varied slightly between samples depending on slight over-recruitment or removing participants for duplicate IP addresses or technical issues). An additional 26 participants were excluded due to duplicate IP addresses within and between surveys (we retained the earliest response), and one participant was excluded for indicating that the video did not work. In terms of the effect of stimulus medium, sensitivity power analyses showed that we had 80% power to detect effect sizes of at least *d* = 0.24, 90% power to detect effect sizes of at least *d* = 0.27, and 95% power to detect effect sizes of at least *d* = 0.31 (two-tailed, α = .05).

#### Materials and procedure

We told the participants that the study concerned how people form their first impressions of others involved in different situations. They were informed from the outset that their participation might involve watching a brief video clip of an assault and robbery and may therefore be somewhat distressing or uncomfortable. They were asked to not participate if they felt they would find this upsetting.

Participants were randomly assigned to view either a real-life CCTV video of a robbery/assault, or read a short vignette that accurately described the events occurring in the CCTV video. Study 2 used four real episodes of robbery/assault taken from youtube.com. In the “elevator mugging” scenario, the video (34 s) showed a woman having her bag snatched by a lone male passenger as she exited an elevator. In the “street attack” scenario, the video (20 s) showed a violent and apparently unprovoked assault of a woman by a female assailant on a busy downtown street near a greengrocer. In the “scooter attack” scenario, the video (16 s) showed a violent attempted mugging of a man on a busy downtown street by a male assailant who escaped on a motor scooter. In the “store robbery” scenario, the video (33 s) showed an attempted robbery of a grocery store during which a male checkout assistant was physically assaulted by a male robber armed with a shotgun. We created text-based versions of each scenario that verbally described, in third person, the content of the video (the data and materials for all studies are available at osf.io/a5zcp). For example, for the “scooter attack” scenario, participants read,

Imagine the scene of a busy downtown street. A motor-scooter with a driver and a passenger pulls to the side of the street. The passenger gets off the scooter and runs up behind a man looking in a store window. The passenger of the scooter grabs the man by his backpack, attempting to steal it. The man resists but is forcefully thrown to the ground and dragged along the sidewalk for a couple of yards. The passenger of the scooter then repeatedly kicks the man in the face before letting go of the bag and running off toward the scooter to make a get-away.

After watching one of the four videos or one of the four text descriptions, participants rated their impression of the victim in both absolute (“How negative-to-positive would you evaluate the robbery/assault victim as a person”; 0 = *very negatively*, 10 = *very positively*) and relative terms (“How negative-to-positive would you evaluate the robbery/assault victim as a person compared to how negative-to-positive you would evaluate yourself as a person”; 0 = *much more negatively than me*; 10 = *much more positively than me*). Finally, except for the “scooter attack” survey, participants provided their age and gender, and responded to an item checking whether the video played successfully, specifically: “If you were asked to watch a video, did it play/work for you ok” (*yes, no*, or *not applicable*).

### Results

Absolute and relative character ratings were recoded to 1–11 for analysis (and rescaled so higher values indicate less favorable impressions of the victim’s character). Descriptive statistics by condition for the individual scenarios and with the data collated across scenarios appear in Table 7. Absolute and relative character ratings were submitted to a 4 (scenario) × 2 (medium: video vs. text) × 2 (rating type: relative vs. absolute) mixed analysis of variance (ANOVA), with repeated measures on the last factor. Significant main effects of type of rating, *F*(1, 553) = 49.88, *p* < .001, $\eta_{p}^{2}$ = .08; medium, *F*(1, 553) = 3.99, *p* = .046, $\eta_{p}^{2}$ = .01; and scenario, *F*(3, 553) = 3.35, *p* =.02, $\eta_{p}^{2}$ = .01, were qualified by a significant Medium × Type of Rating interaction, *F*(1, 553) = 5.38, *p* = .02, $\eta_{p}^{2}$ = .01 (see bottom row of Table 6). Whereas absolute character ratings of the victim were not significantly different between the video and text conditions, *t*(558.62) = 0.51, *p* = .61, *d* = 0.04, 95% CI of *d* [−0.21, 0.29], relative character ratings were more negative in the video condition compared with the text condition, *t*(552.40) = 3.45, *p* < .001, *d* = 0.29, 95% CI of *d* [0.12, 0.45] (here and throughout, degrees of freedom were Welch corrected where applicable). No other interaction effects achieved statistical significance (all *p*s > .08). Regression analyses of the effect of stimulus medium on relative and absolute character ratings adjusting for the alternate rating type led to the same conclusions (see supplementary materials for details). In sum, more emotionally impactful stimuli (i.e., videos) led to greater victim derogation than less impactful stimuli (i.e., text vignettes), but only when gauged using relative (vs. absolute) scales.

**Table 6.** Descriptive Statistics for Relative and Absolute Ratings of the Victim’s Character Across Scenarios by Type of Medium.

Scenario	Text		Video	
	Absolute	Relative	Absolute	Relative
Elevator (*n* = 138)	5.86 (2.61)	6.33 (1.81)	5.36 (2.81)	6.80 (2.14)
Street (*n* = 142)	5.82 (2.44)	6.13 (1.67)	5.86 (2.29)	6.68 (1.73)
Scooter (*n* = 143)	5.76 (3.10)	6.25 (2.24)	6.96 (2.94)	7.28 (2.14)
Robbery (*n* = 138)	5.49 (2.76)	6.06 (1.96)	5.19 (2.89)	6.33 (2.36)
Collated	5.74 (2.73)	5.88 (2.82)	6.19 (1.93)	6.78 (2.12)

*Note.* Higher values indicate greater derogation of the victim’s character.

## Study 3

In Study 2 we found that on average victimization contexts that were more distressing and psychologically arousing for observers increased relative victim derogation. One issue is that these findings cannot speak directly to the role that perceived injustice plays in the derogation of innocent victims under conditions of high and low emotional impact, as we only used scenarios where the victim was presumed to suffer through little fault of their own (i.e., was innocent), and we did not otherwise attempt to manipulate perceived injustice. In our meta-analysis, we included studies that varied the injustice of the situation (e.g., the extent of a victim’s suffering) and found that emotional impact (vis-à-vis stimulus medium) modulated effect sizes. Although Study 2 provided important evidence for increased victim derogation when the context was more (vs. less) emotionally impactful, it is not clear how much the injustice of the victimization context matters for victim derogation to manifest under conditions of high and low emotional impact. In Study 3, then, we adopted a moderation-of-process design (Spencer et al., 2005) to examine the role that injustice plays in the effect of emotional impact on victim derogation by manipulating the innocence of the victim along with varying the stimulus medium (video vs. text).

Several studies have shown that observers perceive the suffering of innocent victims as more unfair and unjust than the suffering of non-innocent victims (e.g., Correia et al., 2007; Harvey et al., 2014). Lerner’s (2003) theorizing suggests that the emotional impact of a victimization context should affect victim derogation more strongly when an innocent victim suffers than when a non-innocent victim suffers, as the suffering of an innocent (vs. non-innocent) victim poses a greater threat to the need to believe in a just world. Put differently, insofar as conditions of high emotional impact translate perceived injustice into a stronger motivational imperative to defend the need to believe in a just world, the effect of victim innocence on character ratings of the victim is likely magnified under conditions of high (vs. low) emotional impact.

In Study 3, participants either viewed a CCTV video of an assault and attempted robbery or read a text-based vignette describing the same scenario. Crossed with this manipulation, participants learned (or did not learn) that the victim brought about his own suffering. Based on the foregoing analysis and the results of our meta-analysis, we expected that participants would devalue the victim’s character when the context was more (vs. less) emotionally impactful, but only when the victim was innocent. Like Study 2, we assessed both relative and absolute character ratings of the victim.

### Method

#### Participants

A total of 801 participants (50% female; *M*_age_ = 35.08, *SD*_age_ = 11.54) were recruited online via Amazon’s Mechanical Turk. An additional seven participants were excluded due to duplicate IP addresses (we retained the earliest response), and a further 14 participants were excluded for incorrectly answering an attention check (described below). The required number of participants was fixed ahead of data collection. Sensitivity power analyses showed that we had 80% power to detect effect sizes of at least *d* = 0.20, 90% power to detect effect sizes of at least *d* = 0.23, and 95% power to detect effect sizes of at least *d* = 0.26 (two-tailed, α = .05).

#### Materials and procedure

Like Study 2, in Study 3 participants were randomly assigned to either view a CCTV video of an assault/robbery or read a short vignette that described the events occurring in the video. All participants received the “scooter assault” scenario from Study 2. We used only this scenario for Study 3 because it was deemed the most straightforward and plausible to manipulate the innocence of the victim compared with the other scenarios we used in Study 2.

Half of the participants then read a short passage of text, presented on a separate page, which formed our manipulation of victim innocence. Following Callan et al. (2014), participants in the non-innocent condition were informed that

A local news report about the incident you just reviewed revealed that the individual who was robbed and assaulted was a local drug dealer. The men on the scooter were members of a rival gang and were attempting to steal illicit drugs that were discovered on the victim.

Participants in the innocent victim condition received no additional information and instead advanced immediately to the dependent measures. Participants then rated their impression of the victim in both absolute and relative terms as per Study 2, and those in the non-innocent condition completed an attention check, specifically “What was the robbery/assault victim described as?” (*a fraudster; a drug dealer; a tourist; a window cleaner*). Finally, participants provided their age and gender, and responded to an item checking whether the video played successfully: “If you were asked to watch a video, did it play/work for you ok” (*yes; no; not applicable*).^5^

### Results and Discussion

Absolute and relative character ratings were recoded 1 to 11 (higher values indicate greater victim derogation) and submitted to a 2 (medium: text vs. video) × 2 (victim innocence: innocent vs. non-innocent) × 2 (measure type: absolute vs. relative) mixed-design ANOVA, with repeated measures on the last factor. There was a statistically significant main effect of measurement type, indicating that the absolute ratings of the victim’s character were more favorable (*M* = 6.81, *SD* = 2.73) than were relative ratings (*M* = 7.47, *SD* = 2.35), *F* (1, 797) = 78.73, *p* < .001, $\eta_{p}^{2}$ = .09 (cf. Dawtry et al., 2018). There were also significant main effects of medium, *F*(1, 797) = 4.88, *p* = .029, $\eta_{p}^{2}$ = .01, and victim innocence, *F*(1, 797) = 178.35, *p* < .001, $\eta_{p}^{2}$ = .18.

Contrary to our expectations and Study 2 findings, there were no statistically significant interactions involving type of ratings (all *p*s > .19), suggesting that the effects of innocence and medium and their interaction were statistically equivalent across type of ratings. As shown in Table 7, collapsing across type of ratings, there was a statistically significant Medium × Victim Innocence interaction, *F*(1, 797) = 10.96, *p* < .001, $\eta_{p}^{2}$ = .014. Ratings of the non-innocent victim’s character were not significantly different between the text and video scenarios, but ratings of the innocent victim’s character were more negative in the video compared with the text condition (see Table 7; statistical details for these comparisons are presented in the supplementary materials). Looking at this interaction from a different angle, ratings of the innocent victim’s character converged more toward ratings of the non-innocent victim’s character under high emotional impact (i.e., became relatively more negative), than they did under low emotional impact. What this pattern suggests is that under high just-world threat (i.e., being exposed to the assault/robbery of an *innocent* victim), observers’ ratings of the victim’s character accord more with observers’ ratings of someone who was objectively a “bad” person and brought about his own suffering (i.e., a drug dealer) under high (vs. low) emotional impact. Of course, non-innocent victims will almost always be rated less favorably than truly innocent victims (see Dawtry et al., 2018; Harvey et al., 2014), but under higher emotional impact, participants’ ratings of the innocent victim’s character crept toward character ratings of the victim who was objectively foolish, irresponsible, and otherwise morally suspect.

**Table 7.** Means (Standard Deviations) for Relative and Absolute Ratings of the Victim’s Character by Medium and Victim Innocence.

	Text			Video		
Condition	Relative	Absolute	Marginal means	Relative	Absolute	Marginal means
Innocent	6.07 (2.04)	5.45 (2.90)	5.76_a_ (2.25)	6.83 (2.15)	6.31 (2.90)	6.16_b_ (2.33)
Non-innocent	8.61 (2.01)	7.82 (2.06)	8.21_c_ (1.72)	8.41 (2.20)	7.68 (2.22)	8.05_c_ (1.95)

*Note.* Higher values indicate more negative character ratings of the victim. Marginal means (i.e., averaged across relative and absolute ratings within victim innocence conditions) that do not share a common subscript are statistically significantly different (*p* < .001).

These results also help to address one potential limitation of Study 2; specifically, that the video and text-based scenarios differed along basic structural dimensions that could have led to differences in evaluations of the victim. For example, text-based vignettes can never fully represent details of a victimization context in the same way that videos can (e.g., exact facial expressions and body posture are presumably more richly and accurately conveyed visually), which perhaps provides viewers with more contextual details to form their impressions of the victim. There are also basic differences in how perceivers mentally engage with information presented as text compared with video (e.g., reading speed, use of mental imagery, reading proficiency, and comprehension; Beentjes & van der Voort, 1993; Furnham et al., 2002; Rockwell & Singleton, 2007) that could potentially affect observers’ ability to comprehend the episode. However, because we observed an effect of stimulus medium on victim derogation only when the victim was innocent (i.e., an interaction pattern), it is unlikely that these basic differences in the modality of presentation were driving our effects—otherwise, we would have expected the medium to affect victim derogation regardless of the victim’s innocence, because such differences in modalities were present across both of the innocence conditions. Instead, we found that the effect of stimulus medium on victim derogation was modulated by victim innocence, such that participants derogated the victim to a greater extent under high (vs. low) emotional impact but only under conditions of just-world threat.

## General Discussion

Across a meta-analysis and primary studies, we provide converging evidence that the emotional impactfulness of the victimization context for observers enhances their derogation of the innocent victim’s character. Our meta-analytic findings revealed a small overall victim derogation effect, which was modulated by emotional impact: studies that employed more emotionally impactful stimuli reported larger victim derogation effects. One issue with this finding was that emotional impact at the level of studies was confounded with year of appearance, such that older studies tended to also use more emotionally impactful contexts (i.e., that were more vivid, ostensibly real, and proximal). Thus, we were unable to draw strong conclusions about the role of emotional impact over and above the influence of year of appearance from our meta-analytic findings alone. To address this issue empirically, in Studies 2 and 3, we experimentally manipulated the emotional impactfulness of victimization scenarios via the stimulus medium by which they were presented to participants (i.e., text vignettes vs. CCTV footage). In Study 2, relative (but not absolute) impressions of the victim’s character were more negative when the events were shown as CCTV footage than when they were described in text form. That is, victim derogation was higher when participants were exposed to a victim via high (vs. low) emotionally impactful stimuli. Our supplementary studies confirmed that our manipulation of stimulus medium affected emotional impact, with scenarios presented via CCTV eliciting more negative affect and psychological arousal than those presented via text vignettes.

Finally, in Study 3, we examined the effect of emotional impact under conditions of high versus low threat to the need to believe in a just world. When the victim was non-innocent (low threat), derogation was similar regardless of whether the victim was presented via a high- (i.e., CCTV) or low-impact (i.e., text) stimulus medium. When the victim was innocent (high threat), however, derogation was higher when the victim was presented via a high (vs. low) impact medium. In sum, high-impact stimuli only produced greater victim derogation under conditions in which, according to just-world theory, defensive victim derogation should occur—that is, when the victim was innocent and thus represented a greater threat to their faith in a just world.

### Implications

#### Theoretical implications

Just-world theory was borne from experimental research in the 1960s discovering that observers may be threatened enough by an innocent victim’s suffering to devalue their character. Contemporary research, however, has found inconsistent evidence for the victim derogation effect, casting in doubt one of the phenomena most associated with just-world theory. Reflecting on nearly 40 years of research in the field, Lerner (2003) lamented that because of this reliance on using less impactful stimuli in contemporary research, social psychologists had “lost” the justice motive. Our meta-analysis confirmed that the size of the victim derogation effect has indeed declined since Lerner and Simmons (1966) and suggests that one reason for this decline has been an increased reliance on “low impact” victimization contexts in contemporary research. The results of our two primary studies more definitively provide the first empirical support for Lerner’s (2003) theoretical contention that emotionally impactful injustices elicit greater victim derogation than those that do not provoke a stronger emotional response, highlighting that emotional arousal is an important, if not necessary, component of the phenomenon.

Why, though, does emotional impact underpin victim derogation in this way? Broadly speaking, emotional arousal tends to affect people’s capacity and motivation to thoughtfully consider their immediate social contexts (Bodenhausen, 1993; Strack & Deutsch, 2004), and therefore enhances the use of more stereotypic responses and evaluative associations. Although not examined in the current research, Lerner and others have argued that reactions toward victims occur effortlessly and intuitively, automatically triggering in response to familiar situational cues (Hafer & Bègue, 2005; Lerner, 1998, 2003; Lerner & Clayton, 2011; Lerner & Goldberg, 1999). According to Lerner (2003, p. 389), these schema-based reactions entail “simple univalent associations of outcomes, personal characteristics, emotions and restorative acts” (e.g., “bad things happen to bad people”), and promote responses which satisfy the motivation to defend one’s commitment to a just world in the face of contrary evidence. High emotional arousal elicited from a “high impact” victimization context (e.g., CCTV footage of actual victimizations) may therefore interfere with an observer’s ability to thoughtfully appraise the circumstances surrounding an injustice and adjust their initial, intuitive, negative reactions to align with social norms and conventional rules for assigning blame and deserving.

Conversely, in less emotionally engaging situations, motives besides defending justice beliefs may take higher priority, and people presumably possess greater capacity to engage in a thoughtful, considered appraisal of the circumstances at hand. Emotionally detached observers may be relatively more concerned with managing their impressions, and behaving in normatively appropriate and fair-minded ways. As such, their responses will likely be framed in terms of social norms and conventional rules of deserving (Lerner, 2003). Insofar as social norms hold that innocent victims should be treated with sympathy and not derogated for their misfortune, devaluing a victim’s character risks appearing callous, perhaps to the self as well as others (Dawtry et al., 2018). Thus, emotionally disengaged observers are less likely to manifest counter-normative and seemingly irrational responses, such as victim derogation.

Just as emotionally impactful contexts make victim derogation more likely, so too do relative (vs. absolute) ratings of a victim’s character. In Study 2, we found that the effect of stimulus medium on victim derogation occurred when participants made their ratings in relative rather than absolute terms. Drawing on Dawtry et al.’s (2018) research, we reasoned that, because relative measures are less prone to the influence of social norms or personal standards that mute the overt expression of negative attitudes toward innocent victims, they can be expected to reveal more negative evaluations of an innocent victim’s character. In Study 3, however, although relative evaluations of the victim were less favorable in general, measurement type did not interact, independently or in concert, with either stimulus medium or victim innocence, lending a note of caution to this interpretation. It is worth highlighting that the victimization scenario we used in Study 3 (“scooter attack”) showed the weakest stimulus Medium × Measurement Type interaction effect in Study 2 (see Table 6), so there might be some peculiar feature of this context that leads to enhanced derogation across *both* rating types. One such feature might be the sheer brutality of the victimization in this scenario (i.e., a man on the ground being kicked in the face) that provokes a derogatory response across ratings compared with the other contexts we used in Study 2 (e.g., a victim’s bag being snatched). Nevertheless, given our Study 2 findings and the findings of Dawtry et al. (2018), future research would benefit from the inclusion of both absolute and relative character ratings to further explore their respective influences on the derogation of innocent victims.

#### Methodological implications

Researchers have commented that revealing experimental evidence for the rejection of victims in the face of just-world threat is not easy (e.g., van den Bos & Bal, 2016), and our results support this sentiment: In Studies 1 and 3, only when the stimuli were emotionally impactful did participants devalue the victim under just-world threat. The current work therefore casts important new light on the situations that constrain and enhance the tendency for people to derogate an innocent victim, and they provide direction for researchers interested in further exploring the causes, consequences, and moderators of the victim derogation effect. Specifically, the results of our meta-analysis and Supplementary Study 1 suggest that victimization contexts that are vivid, real, or ostensibly real, and spatiotemporally proximal are more emotionally impactful than those that lack any one of these attributes. Therefore, researchers interested in garnering evidence for the importance of a just world to people in the face of just-world threat should consider ways of developing and using stimuli that are more motivationally and emotionally engaging for observers. In the current studies, we explored how using video (vs. text-based) portrayals of victimization contexts provides a practically straightforward way of depicting events as vivid and real, but this is by no means the only way of doing so. Indeed, people can be deeply moved by instances of harm-doing and injustice that they read about in the news. Although text-based and presumably less vivid, such episodes of injustice are nonetheless real and immediate and can thus be emotionally impactful. These situations stand in stark contrast to the kinds of stimuli used by contemporary researchers where little effort has gone into portraying events as real, immediate, and vivid (including our own work, for example, Harvey et al., 2014).

#### Practical implications

Beyond the theoretical and methodological contributions of the current research, the findings we report here are also of practical importance because they shed light on the contexts where victim derogation is more likely to manifest, not only in the context of research, but also in the real-world. This is important because, as a form of secondary victimization (Condry, 2010), negative social reactions toward victims can compound an individual’s experiences of injustice, for example, through others’ reduced willingness to alleviate their suffering, withdrawal of effective social support, or negatively tinged social interactions (Herbert & Dunkel-Schetter, 1992; Koper et al., 1993). Research and theorizing on interactional justice, for example, suggests that insensitive or disparaging communications from others can negatively impact upon a victim’s self-esteem, thus exacerbating the psychological harm of victimization (Koper et al., 1993; Tyler et al., 1996).

One important real-world context to consider in relation to secondary victimization is the various stages of criminal justice processes—a victim’s interactions with police, judges, and other legal professionals (e.g., Orth, 2002). Due to their involvement in interviewing victims, undertaking court proceedings, and so on, these professionals are regularly exposed to *real* victims, in an immediate, vivid, and emotionally intensive way, precisely the conditions that our findings suggest are most likely to elicit victim derogation. Our findings suggest that, to reliably examine the extent and conditions under which forms of secondary victimization occur in criminal justice contexts, experimental researchers should aim to replicate these emotionally impactful conditions as closely as possible, or seek to examine these contexts directly in the real-world.

### Limitations and Future Research Directions

We suggested that the medium of presentation—for example, video, text, or still images—provides an objective proxy for the emotional impact of an injustice context, insofar as it partially determines other psychologically relevant attributes of the stimuli, such as vividness (the intensity with which injustice is depicted) and veracity (whether suffering is believed to be real or plausible). Relatively little research has directly compared the emotional impact of video versus text stimuli, as we did in our primary studies, although existing findings support our general line of reasoning. Video and photos, for example, have been shown to elicit stronger self-reported emotion than text-based descriptions alone (Bright & Goodman-Delahunty, 2006; Koehler et al., 2005; Yadav et al., 2011), and within media relying on the same perceptual modalities, subtle changes such as showing a video in 3D (vs. 2D; Rooney et al., 2012) or in a virtual reality environment (vs. 3D; Visch et al., 2010) can positively impact observers’ emotions.

These effects are often explained in terms of the vividness, veracity, and proximity of different mediums, albeit often using different terms (e.g., Geen, 1975; Mendelson & Papacharissi, 2007; Rooney et al., 2012; Slater & Wilbur, 1997; Visch et al., 2010). Research further suggests that these attributes are *independently* related to the intensity of emotion evoked by various stimuli, including those depicting harm, suffering, or violence, as gauged by both self-reported emotions and physiological indices of emotional arousal (e.g., Codispoti & De Cesarei, 2007; Gu & Han, 2007; Mendelson & Papacharissi, 2007; R. F. Simons et al., 1999). Because, in our meta-analytic data and primary studies, these attributes were positively interrelated via the medium—for example, compared with text, CCTV is more vivid, real, and proximal—the present data say relatively little about the independent contribution of each to overall levels of emotional impact and victim derogation. Future research could seek to isolate and orthogonally manipulate vividness, veracity, or proximity *within* mediums to better delineate the relative contribution of each to observers’ reactions to victimization contexts. Vividness, for example, could perhaps be manipulated directly by varying the resolution or coloring of videos or still images of episodes of victimization (e.g., Bradley et al., 2001), and cues as to the origin of stimuli can easily be varied to influence perceptions of veracity (e.g., Lerner, 1971; C. W. Simons & Piliavin, 1972).

Of course, emotional impact is also likely influenced by a variety of other factors, such as the nature of the injustice (e.g., a victim suffering status, punishment outcome) and how someone was victimized (e.g., mundane misfortune, sexual assault). As Hafer and Bègue (2005) observed in their review of the just-world literature, researchers have used a variety of different operationalizations of injustice manipulations, and the contexts for the injustice have been similarly varied. This poses challenges for determining the extent to which emotional impact, and in turn victim derogation, rely on specific contextual features of the victimization itself, such as the cause of a victims suffering (e.g., illness, violence, mundane misfortune). As shown in Table 2, across the 55 studies we included in our meta-analysis, there were 23 unique combinations of the injustice manipulation used and the context for the injustice (e.g., someone’s illness was less or more severe). Indeed, the large variability in the causes of victims’ suffering represented in the papers included in the meta-analysis prevented us from reliably examining its role in emotional impact, and whether it moderated victim derogation.

As well as features of the victimization context, emotional responses toward injustice, may be influenced by the nature of the relationship between victim and observer, such as their social distance—people may react more strongly toward victims with whom they share common attributes and identity, such as ethnicity (Aguiar et al., 2008; Correia et al., 2007), age (Callan et al., 2012), or gender (Drout & Gaertner, 1994). Indeed, research suggests that the suffering of out-group members elicits a dampened emotional response compared with in-group suffering (Batson & Ahmad, 2009; Cikara et al., 2011). For example, physiological indices of emotional arousal and activation in brain areas related to emotional or pain processing (e.g., the anterior cingulate cortex) are attenuated when observing the suffering of an out-group (vs. in-group) member (Azevedo et al., 2013; Mathur et al., 2010; Xu et al., 2009). Relatedly, research suggests that in-group victims are more threatening to the need to believe in a just world (Aguiar et al., 2008; Correia et al., 2007). This perhaps reflects that observing the suffering of others who are similar (vs. dissimilar) to the self can provoke a stronger emotional response. We were not, however, able to examine this in the meta-analysis because few papers systematically varied victim–participant similarity (e.g., comparing an out-group vs. in-group victim), and there was generally no reliable means for us to judge the extent to which victims and participants were similar.

Other factors open to further investigation are the roles that individual and cultural differences play in modulating the effect of high- versus low-impact contexts on victim derogation. Although there is limited published research on individual difference moderators of the effects of threats to just-world beliefs on defensive responses (for exceptions, see Hafer & Rubel, 2015), theoretically one might expect those individuals with a propensity to defend their justice beliefs in the face of threat, such as individuals who place greater importance on pursuing long-term goals (Callan et al., 2013; Hafer, 2000b) or who are higher in self-reported just-world beliefs (Hafer & Sutton, 2016), to show greater victim derogation when exposed to high- versus low-impact episodes of victimization. Furthermore, since Lerner and Simmons (1966), most, if not all, research on the victim derogation effect has been conducted using Western samples, which limits the generalizability of our findings. Given that culture influences people’s perceptions of justice and injustice (see Fischer, 2016), it will be important for future research to examine the degree to which different cultural values and dynamics modulate the victim derogation effect vis-à-vis the emotional impactfulness of the context for observers.

A further important avenue for future research concerns the role of emotional impact in other justice-motivated responses to innocent victims, such as victim blaming. Blame and derogation are similar insofar as either can reflect a motivated attempt to rationalize injustice and defend the need to believe in a just world, and research has shown that they are indeed moderately correlated (e.g., Harvey et al., 2014). Yet, they are conceptually different—derogation entails finding fault with a victim’s *character*, whereas blaming entails finding fault with their *behavior* (for discussions of this distinction, see Janoff-Bulman, 1979; Karuza & Carey, 1984; Lerner & Miller, 1978). A person’s bad character cannot directly cause them to suffer or vice versa, yet a victim’s behavior may often be plausibly linked to their suffering. Hence, unlike derogation, victim blaming need not stem from a motivated attempt to *rationalize* an injustice, but can instead reflect a relatively more rational (if, perhaps, underdeveloped, biased, or misinformed) attempt to *causally explain* how an injustice occurred. Our reasoning and the present findings could suggest that, if or when victim blaming *does* reflect motivated rationalization in service of defending the need to believe in a just world then, like derogation, it should occur more strongly under more (vs. less) emotionally impactful victimization contexts.

Finally, in the present work, emotional impact was measured via self-report only—participants forecast their emotional response to a range of hypothetical stimuli (Study 1), or reported the level of negative affect and psychological arousal they experienced when exposed to an episode of victimization in video or text form (Supplementary Studies 2a and 2b). Future research should seek to corroborate our findings by employing physiological indices of emotional experiences, such as electrodermal activity, pupil dilation, or brain responses related to empathy (Bernhardt & Singer, 2012). Although much research shows that more vivid, proximal, and realistic stimuli elicit greater physiological arousal (e.g., Codispoti & De Cesarei, 2007; Gu & Han, 2007; R. F. Simons et al., 1999), as well as self-reported emotion, it is not clear whether physiological indices of arousal are related to evaluations of victims, as our reasoning would suggest.

### Conclusion

In summary, we found that the derogation of an innocent victim increases under higher (vs. lower) emotionally impactful contexts for observers. This was true of our meta-analysis and our primary experiments. Our meta-analysis, for the first time, showed empirically that the victim derogation effect has declined since Lerner and Simmons’s (1966) seminal study—a decline that we show stems, in part, from the increased use of less emotionally impactful contexts in contemporary research. We speculate that this increased use of less impactful contexts in research has occurred for at least two reasons: (a) the relative ease and cost effectiveness of employing vignette-based victimization scenarios, rather than elaborately staged and ostensibly real episodes of victimization like Lerner and Simmons’s (1966) experiment and (b) increased sensitivities to ethical issues in experimental research involving deception and harm-doing (see, for example, Benjamin & Simpson, 2009). Whatever the reason, the present work provides support for Lerner’s (2003) contention that observers are more likely to devalue an innocent victim’s character under conditions of high emotional impact—the very conditions that are likely closest to contexts where we encounter threats to our need to believe in a just world in everyday life. Despite these advances, it will be important for future research to explore the generality of our findings to other situational and individual difference factors that might play a role in observers’ responses to victimization.

## Supplemental Material

Supplementary_Materials – Supplemental material for Victims, Vignettes, and Videos: Meta-Analytic and Experimental Evidence That Emotional Impact Enhances the Derogation of Innocent Victims Click here for additional data file. Supplemental material, Supplementary_Materials for Victims, Vignettes, and Videos: Meta-Analytic and Experimental Evidence That Emotional Impact Enhances the Derogation of Innocent Victims by Rael J. Dawtry, Mitchell J. Callan, Annelie J. Harvey and Ana I. Gheorghiu in Personality and Social Psychology Review

## Notes

1. As described in the Study 1 Method, where injustice was manipulated across more than two levels, we combined similar conditions or took the average effect across comparisons, and selected specific simple effects for four of 25 factorial studies. To examine the sensitivity of the overall effect to the procedures we used to determine appropriate study-level effects, we fit additional models using non-combined and simple effects. Specifically, we used the smallest versus largest pairwise/simple study-level effect size available (we continued to use main effects for 13 factorial studies for which simple effects could not be computed). These models estimate the lower and upper bound of the overall victim derogation effect, irrespective of the criteria we used to determine effect sizes in the focal analyses. Including only the smallest available study-level effect sizes, the overall victim derogation effect was *d* = 0.08 (*SE* = 0.07), *p* = .268, 95% confidence intervals (CIs) [−0.06, 0.21]; *Q*(54) = 233.49, *p* < .001, τ^2^ = 0.19 (*SE* = 0.05), 95% confidence interval (CI) [0.13, 0.38]. Including only the largest study-level effect sizes, the overall victim derogation effect was *d* = 0.18 (*SE* = 0.06), *p* = .002, 95% CIs [0.07, 0.29]; *Q*(54) = 189.89, *p* < .001, τ^2^ = 0.13 (*SE* = 0.04), 95% CI [0.09, 0.31]. In a model including only main effects across all 25 factorial studies (all other study-level effects were as described in the Method), the overall victim derogation effect was *d* = 0.14 (*SE* = 0.06), *p* = .028, 95% CIs [0.01, 0.26]; *Q*(54) = 228.25, *p* < .001, τ^2^ = 0.15 (*SE* = 0.04), 95% CI [0.11, 0.32].

2. A model including emotional impact scores, year of publication, vividness, and proximity as moderators yielded a significant overall model fit, *Q_M_*(4) = 19.60, *p* < .001, but none of the individual moderators were statistically significant (all *p*s > .08). We did not include veracity as a moderator in these analyses because of the two missing values and different effect size estimates for two of the studies with the coding for veracity (see Study 1 Method).

3. It is worth noting that none of the studies in our meta-analysis used measures that asked participants to evaluate a victim’s character relative to a comparative referent as per Dawtry et al. (2018). Thus, we could not examine relative versus absolute ratings as a possible moderator of victim derogation in our meta-analysis.

4. Questions soliciting participants’ age and gender were inadvertently omitted from one of the surveys (*n* = 143), so the descriptive statistics reported here are from a subsample of the total participants.

5. An additional manipulation validation study found that participants who learned that the victim was innocent perceived the assault and attempted robbery as more unfair than participants who learned that the victim was non-innocent (see Supplementary Study 3 in the supplementary materials).
