# Supplementary material for: Victims, Vignettes, and Videos: Meta-Analytic and Experimental Evidence That Emotional Impact Enhances the Derogation of Innocent Victims
Source: Pers Soc Psychol Rev. 2020 Apr 22;24(3):233–59. doi: 10.1177/1088868320914208 (PMC7506872; doi:10.1177/1088868320914208)
Supplement: Supplementary_Materials – Supplemental material for Victims, Vignettes, and Videos: Meta-Analytic and Experimental Evidence That Emotional Impact Enhances the Derogation of Innocent Victims [file Supplementary_Materials.pdf]

Supplementary Materials for

Victims, Vignettes, and Videos: Meta-Analytic and Experimental Evidence that Emotional Impact Enhances the Derogation of Innocent Victims

Contents

SUPPLEMENTARY STUDY 1: THE EFFECTS OF VIVIDNESS, PROXIMITY, VERACITY, AND SEVERITY ON IMAGINED EMOTIONAL IMPACT 3

SUPPLEMENTARY STUDIES 2A AND 2B: THE EFFECT OF STIMULUS MEDIUM ON EMOTIONAL IMPACT 6

Supplementary Study 2a 6

Supplementary Study 2b 8

SUPPLEMENTARY STUDY 3: INNOCENCE MANIPULATION VALIDATION 10

SUPPLEMENTARY STATISTICAL ANALYSES FOR STUDY 2 11

FOLLOW-UP TESTS FOR THE INNOCENCE X STIMULUS MEDIUM INTERACTION IN STUDY 3 12

REFERENCES 13

SUPPLEMENTARY STUDY 1: THE EFFECTS OF VIVIDNESS, PROXIMITY, VERACITY, AND SEVERITY ON IMAGINED EMOTIONAL IMPACT

In Supplementary Study 1, we systematically varied whether an assault and robbery was high or low in vividness, proximity, veracity, and outcome severity. We told participants (N = 102; 43% female; M_{age} = 29.09, SD_{age} = 10.29; recruited online via Prolific Academic) that the study was investigating people's emotional reactions to unfortunate events. Participants first read an assault/robbery scenario:

> It was 5:15pm. Riley Jordan had just finished work and was walking to the bus stop to go home. As she turned the corner and with her bus stop in sight, a man wearing a black balaclava and hooded top pulled her into an alley off the main street. The man pushed Riley to the ground, snatched her handbag, and ran off.

After reading the scenario, participants were asked to consider how emotionally arousing/impactful/upsetting they would find learning about the robbery and assault when specific details of the situation (e.g., when it occurred) and the medium through which they could have been exposed to it (i.e., video footage or text description) varied. Specifically, participants were presented with 16 different descriptions of conditions surrounding the assault and robbery that varied in (a) veracity (i.e., real or hypothetical), (b) proximity (i.e., occurred yesterday or 20 years ago), and (c) severity (i.e., had minimal or severe consequences for Riley). All possible combinations of these factors were represented across the 16 descriptions. For example, for the high veracity, high vividness, temporally proximal, and severe outcome description, participants read: "You watched a real CCTV video of the actual robbery. The robbery occurred yesterday. Riley was hospitalised with a fractured skull from being pushed to the ground." For the low veracity, low vividness, temporally distant, and minimal outcome description participants read: "You read a text description of a hypothetical robbery. The robbery occurred 20 years ago. Riley had minor bruising from being pushed to the ground."

Participants were asked to imagine how they would immediately feel in response to being exposed to Riley's assault and robbery, in the manner described, for each of the descriptions. Participants then rank ordered the 16 descriptions from most emotionally impactful and upsetting to least emotionally impactful and upsetting. The descriptions were

presented in a random order across participants. We scored the rank orders such that higher scores indicate more emotionally impactful contexts.

The mean rank for each description is shown in Table S1. Participants' rankings of the descriptions were submitted to a 2 (Vividness: video vs. text) X 2 (Veracity: real vs. hypothetical) X 2 (Proximity: yesterday vs. 20 years ago) X 2 (Outcome Severity: severe vs. minimal) repeated measures ANOVA. Analyses revealed significant main effects of Vividness, $F(1, 101) = 93.91$, $p < .001$, $\eta_p^2 = .48$, Veracity, $F(1, 101) = 156.04$, $p < .001$, $\eta_p^2 = .61$, Proximity, $F(1, 101) = 65.01$, $p < .001$, $\eta_p^2 = .39$, and Severity, $F(1, 101) = 61.45$, $p < .001$, $\eta_p^2 = .38$. The two-way Veracity X Severity interaction was the only statistically significant interaction to emerge from these analyses, $F(1, 101) = 4.77$, $p = .003$, $\eta_p^2 = .38$, which suggests that the effect of outcome severity on emotional impact was stronger when the context was real, $t(101) = 8.17$, $p < .001$, rather than hypothetical, $t(101) = 5.79$, $p < .001$. Apart from this lone interaction effect, the effect of vividness, veracity, proximity, and outcome severity on participants' rankings of emotional impact were additive, such that the more the descriptions contained the attributes theoretically associated with emotional impact, the more participants deemed the victimization context as emotionally impactful (see Table S1). Indeed, with description as the basic unit of analysis, as the number of emotionally impactful attributes included in the description increased (which ranged from 0 to 4), the more highly participants ranked them as emotionally impactful on average, $r(14) = .94$, $p < .001$. These results therefore complement those from our meta-analysis by showing that, at least in terms of how participants imagine they would feel in these situations, victimization contexts that are vivid, real, temporally close, or severe are more emotionally impactful relative to contexts that are low in vividness, hypothetical, distal, or less severe.

Table S1

Descriptive statistics for emotional impact rankings by description and the number of attributes associated with emotional impact present.

	**Description**		**Attributes Set**	
	M	***SD***	***M***	***SD***
**All attributes present**			14.40	3.69
Real, video, proximal, severe	14.40	3.69		
**3 attributes present**			11.10	1.92
Real, video, proximal, minimal	11.55	3.46		
Real, video, distal, severe	12.75	3.24		
Real, text, proximal, severe	10.75	3.64		
Hypothetical, video, proximal, severe	9.33	3.95		
**2 attributes present**			8.40	0.82
Real, text, distal, severe	8.87	3.28		
Hypothetical, text, proximal, severe	6.75	4.07		
Hypothetical, video, distal, severe	8.40	3.59		
Real, text, proximal, minimal	8.75	3.63		
Real, video, distal, minimal	9.95	3.61		
Hypothetical, video, proximal, minimal	7.69	3.63		
**1 attribute present**			5.76	1.81
Hypothetical, text, distal, severe	5.10	3.65		
Real, text, distal, minimal	6.95	3.23		
Hypothetical, text, proximal, minimal	4.75	3.75		
Hypothetical, video, distal, minimal	6.24	3.62		
**No attributes present**			3.76	4.14
Hypothetical, text, distal, minimal	3.76	4.14		

Note. Higher values indicate lower rankings of impactfulness.

SUPPLEMENTARY STUDIES 2A AND 2B: THE EFFECT OF STIMULUS MEDIUM ON EMOTIONAL IMPACT

In Supplementary Study 1, we found that participants ranked victimization contexts that were more vivid, real, temporally close, and severe as more emotionally impactful. Although consistent with our conceptual analysis, one issue with these findings is that participants *imagined* how they would feel across various substantiations of the same victimization context rather than reporting their emotional experiences after actually observing someone being victimized. It is well-established that people often make affective forecasting errors; that is, their predictions about the emotional intensity of an event do not always match their actual emotional experiences of it (Wilson & Gilbert, 2003). From Lerner's (2003) analysis and our Study 1 findings, we assumed that victimization contexts that were more vivid would be experienced as more emotionally impactful, but this assumption has yet to be tested empirically. To this end, in Supplementary Studies 2a and 2b, we tested whether real episodes of victimization presented in video form are more emotionally impactful than their text-based counterparts.

Supplementary Study 2a

In Supplementary Study 2a, we asked participants to make direct comparisons of CCTV versus text-based victimization contexts in terms of their relative emotional impact, arousal, and distress.

Method

Participants. Seventy-nine participants (46% female; M_{age} = 35.68, SD_{age} = 11.09) were recruited online via Prolific Academic. To ensure good comprehension of the material, we recruited only native English speakers residing in the UK or US. An additional three participants were excluded due to duplicate IP addresses (we retained the earliest response), and a further two participants were excluded for indicating that at least one of the videos did not work. Participants were told that they would be watching videos of crimes and were asked to not participate if they believed this would be upsetting. Sensitivity power analysis showed that we had 80% power to detect a "small-to-medium" effect (dz = 0.32) of the relative emotional impact of text versus video presentations (two-tailed, α = .05).

***Materials and Procedure*.** Supplementary Study 2a used four real episodes of assault/robbery taken from youtube.com. In the "elevator mugging" scenario, the video (34s) showed a woman having her bag snatched by a lone male passenger as she exited an elevator. In the "street attack" scenario, the video (20s) showed a violent and apparently unprovoked assault of a woman by a female assailant on a busy downtown street near a greengrocer. In the "scooter attack" scenario, the video (16s) showed a violent attempted mugging of a man on a busy downtown street by a male assailant who escaped on a motor scooter. In the "store robbery" scenario, the video (33s) showed an attempted robbery of a grocery store during which a male checkout assistant was physically assaulted by a male robber armed with a shotgun. We created text-based versions of each scenario that verbally described, in third-person, the content of the video (the data and materials for all studies are available at osf.io/a5zcp). For example, for the "scooter attack" scenario, participants read:

> Imagine the scene of a busy downtown street. A motor-scooter with a driver and a passenger pulls to the side of the street. The passenger gets off the scooter and runs up behind a man looking in a store window. The passenger of the scooter grabs the man by his backpack, attempting to steal it. The man resists but is forcefully thrown to the ground and dragged along the sidewalk for a couple of yards. The passenger of the scooter then repeatedly kicks the man in the face before letting go of the bag and running off toward the scooter to make a get-away.

Adopting a Latin-square design, in each of two trials, participants were randomly assigned to view one of the four videos (or text scenarios), followed on a separate page by one of the four text scenarios (or videos; the presentation order of text and videos was randomized). Every participant was exposed to all four scenarios in either text or video form, such that each scenario occurred only once and in one format only, across two separate video-text (or text-video) pairings. All twelve possible video and text scenario combinations were represented, in each of two orders (i.e., video first/text first). In sum, each trial involved watching one video scenario followed by a (non-matching) text scenario, or vice versa.

On each trial, on a separate page immediately following presentation of the video and text scenario, participants responded to three separate comparative items regarding the video and text stimuli pairing they had just seen, specifically "Comparing the CCTV footage versus the text description, which of the two did you find the most emotionally impactful/arousing/distressing" (1 = *CCTV footage much more emotionally impactful/arousing/distressing*; 4 = *Equally emotionally impactful/arousing/distressing*; 7 =

Text description much more emotionally impactful/arousing/distressing). Finally, participants provided their age and gender, and responded to an item checking whether the videos played successfully, specifically: “Did both videos you were shown work/play OK for you?” (*Yes, both worked*; *Only the first worked*; *Only the second worked*; *Neither one worked*).

Results

The three comparative items were averaged to form a composite measure of the relative emotional impactfulness of the video compared to the text stimuli (α = .92). Comparing within-subjects, and collapsing across all 12 scenario combinations, emotional impact was not significantly different depending on whether a video (M = 3.00, SD = 1.72) or text scenario (M = 2.62, SD = 1.52) was presented first, $t(40) = 1.23$, $p = .21$, $d = 0.19$, 95% CI of d [-0.11, 0.51][1]. Collapsing across display order (text then video vs. video then text), trials, and scenario combinations (e.g., scooter vs. elevator; elevator vs. street; etc.), a t-test against the scale midpoint (4) indicated that scenarios were judged as more emotionally impactful when presented in video form relative to text form (M = 3.01, SD = 1.30), $t(78) = 6.77$, $p < .001$, $d = 0.76$, 95% CI of d [0.43, 1.08].

Supplementary Study 2b

Supplementary Study 2b extended and replicated our Supplementary Study 2a findings by employing the Self-Assessment Manikin (SAM; Bradley & Lang, 1994) as a measure of participants’ experienced pleasure and arousal when presented with *either* a CCTV or a text-based victimization scenario. The SAM is a widely used, reliable, and valid method of gauging affective experiences (Huang et al., 2015; Marchewka, Żurawski, Jednoróg, & Grabowska, 2014).

Method

Participants. Seventy-eight participants (34 females; M_{age} = 34.3) were recruited online via Amazon’s Mechanical Turk for a study investigating how people react to different situations involving crimes. An additional eight participants were removed due to duplicate IP addresses (we retained the earliest response), and a further three were excluded for not having completed the survey. No participants indicated that at least one of the videos did

[1] The reduced sample size reflects that approximately half the participants received the same order of text and video in both trials.

not work. Sensitivity power analysis showed that we had 80% power to detect at least a "small-to-medium" effect (*dz* = 0.32) of the stimulus medium manipulation on emotional impact (two-tailed, α = .05)

Materials and Procedure. Supplementary Study 2b used the same four video/text scenarios used in Supplementary Study 2b. Each participant saw all four scenarios; two were in video form while the other two were in text form. Participants were randomly allocated to one of six versions of the study, representing all possible combinations of scenarios and formats. The order in which the scenarios were presented was randomized for each participant.

For each trial, on a separate page following the presentation of the scenario in either video or text form, participants responded to two items of the Self-Assessment Manikin (SAM; Bradley & Lang, 1994) measuring the pleasure (unhappy/annoyed – happy/pleased) and arousal (unaroused/relaxed – aroused/stimulated) they experienced during the scenarios. As per the original SAM (Bradley & Lang, 1994), participants saw five graphic "manikin" representations of the scales and could select any of the figures or points between two figures, resulting in a nine-point rating scale for each dimension. Finally, participants provided their age, gender, and responded to an item checking whether the videos were displayed correctly.

Results

The data were collapsed across scenarios, resulting in four data points per participant, measuring the valence and arousal of video and text trials. A within-subjects *t*-test showed that participants were less happy/pleased when exposed to the video scenarios (*M* = 2.01, *SD* = 1.16) than when they were exposed to the text scenarios (*M* = 2.35, *SD* = 1.31), *t*(77) = 3.14, *p* = .002, *dz* = 0.36, 95% CI of the mean difference = [0.12, 0.56]. As expected, the CCTV scenarios (*M* = 5.70, *SD* = 2.16) were also more psychologically arousing than the text scenarios (*M* = 5.28, *SD* = 2.20): *t*(77) = 2.71, *p* = .008, *dz* = 0.31, 95% CI of the mean difference = [0.11, 0.73].

SUPPLEMENTARY STUDY 3: INNOCENCE MANIPULATION VALIDATION

To validate the innocence manipulation we used in Study 3, we randomly assigned a separate sample of participants from Mechanical Turk (N = 66; 43% female; M_{age} = 34.97, SD_{age} = 13.12; 5 additional participants removed for issues with the video, failing an attention check, or duplicate IP addresses) to either the innocent victim or non-innocent victim condition (all participants viewed the CCTV version of the scenario). Participants rated the degree to which they believed the assault/robbery was unfair and unjust for the victim using 11-point scales that ranged from *very fair* to *very unfair* and *very just* to *very unjust* (the two items were highly correlated, r = .89, p < .001, and averaged to form one measure of perceived injustice). Analyses confirmed that participants rated the assault/robbery as more unjust/unfair for the innocent victim (M = 10.04, SD = 2.21) than for the non-innocent victim (M = 7.94, SD = 2.04), t(63.88) = 4.02, p < .001, d = 0.99.

SUPPLEMENTARY STATISTICAL ANALYSES FOR STUDY 2

In Study 2, a Medium Type X Rating Type ANOVA revealed that the effect of stimulus medium on victim derogation was stronger when participants made their ratings of the victim in relative versus absolute terms. Analyses of the effect of stimulus medium on relative and absolute character ratings adjusting for the alternate rating type led to the same conclusions (i.e., by taking a residualized differences approach). Specifically, regressing relative ratings onto stimulus medium (dummy coded: 1 = video, 0 = text) and absolute ratings yielded a significant effect of medium on relative ratings, $B = 0.52$, $se = 0.14$, 95% CI for B [0.25, 0.80], $t(558) = 3.74$, $p < .001$. Regressing absolute ratings onto dummy-coded stimulus medium and relative ratings yielded no significant effect of medium, $B = -.32$, $se = 0.19$, 95% CI for B [-0.70, 0.06], $t(558) = -1.65$, $p = .10$ (in fact, the adjusted mean difference, shown as B, controlling for relative ratings was reversed for absolute ratings). Consistent with the medium X type of rating interaction reported in the main text, a comparison of overlapping effect sizes (see Diedenhofen & Musch, 2015) revealed that the effect of stimulus medium was significantly larger for relative ratings ($r = .143$) than it was for absolute ratings ($r = .026$), $Z = 3.02$, $p = .003$ (absolute and relative ratings were significantly correlated, $r = .58$, $p < .001$).

FOLLOW-UP TESTS FOR THE INNOCENCE X STIMULUS MEDIUM INTERACTION IN STUDY 3

Ratings of the non-innocent victim's character were not significantly different between the text and video scenarios, $t(388.87) = 0.88$, $p = .38$, $d = -0.09$, 95% CI of d [-0.28, 0.11], but ratings of the innocent victim's character were more negative in the video compared to the text condition, $t(399.96) = 3.54$, $p < .001$, $d = 0.35$, 95% CI of d [0.15, 0.55]. Looking at the interaction in a different way, ratings of the innocent victim's character converged more toward ratings of the non-innocent victim's character under high emotional impact (i.e., became relatively more negative), $t(385.58) = 6.84$, $p < .001$, $d = 0.68$, 95% CI of d [0.48, 0.89], than they did under low emotional impact, $t(375.90) = 12.30$, $p < .001$, $d = 1.23$, 95% CI of d [1.01, 1.44].

Supplementary Materials References

Bradley, M. M., & Lang, P. J. (1994). Measuring emotion: the self-assessment manikin and the semantic differential. *Journal of Behavior Therapy and Experimental Psychiatry*, *25*(1), 49-59.

Diedenhofen, B., & Musch, J. (2015). cocor: A comprehensive solution for the statistical comparison of correlations. *PloS One*, *10*(4), e0121945.

Huang, J., Xu, D., Peterson, B. S., Hu, J., Cao, L., Wei, N., ... & Hu, S. (2015). Affective reactions differ between Chinese and American healthy young adults: a cross-cultural study using the international affective picture system. *BMC Psychiatry*, *15*(1), 60-66.

Lerner, M. J. (2003). The justice motive: Where social psychologists found it, how they lost it, and why they may not find it again. *Personality and Social Psychology Review*, *7*, 388-399.

Marchewka, A., Żurawski, Ł., Jednoróg, K., & Grabowska, A. (2014). The Nencki Affective Picture System (NAPS): Introduction to a novel, standardized, wide-range, high-quality, realistic picture database. *Behavior Research Methods*, *46*(2), 596-610.

Wilson, T. D., & Gilbert, T. D. (2003). Affective forecasting. *Advances in Experimental Social Psychology, 35*, 345 - 411.
